# Transcriptomic Analysis of the Rainbow Trout Response to Single and Co-Infections with *Myxobolus cerebralis* and *Tetracapsuloides bryosalmonae* at Sites of Parasite Entry

**DOI:** 10.3390/ijms26178148

**Published:** 2025-08-22

**Authors:** Naveed Akram, Reinhard Ertl, Reza Ghanei-Motlagh, Christopher J. Secombes, Mansour El-Matbouli, Astrid S. Holzer, Mona Saleh

**Affiliations:** 1 Division of Fish Health, University of Veterinary Medicine, 1210 Vienna, Austria; naveed.akram@vetmeduni.ac.at (N.A.); rghanei-motlagh@upei.ca (R.G.-M.); mansour.el-matbouli@vetmeduni.ac.at (M.E.-M.); astrid.holzer@vetmeduni.ac.at (A.S.H.); 2 VetCore Facility for Research, University of Veterinary Medicine, 1210 Vienna, Austria; reinhard.ertl@vetmeduni.ac.at; 3 Hoplite Research Lab, Department of Pathology and Microbiology, Atlantic Veterinary College, University of Prince Edward Island, Charlottetown, PEI C1A 4P3, Canada; 4 Huntsman Marine Science Centre, St. Andrews, NB E5B 2L7, Canada; 5 Scottish Fish Immunology Research Centre, School of Biological Sciences, University of Aberdeen, Aberdeen AB24 2TZ, UK; c.secombes@abdn.ac.uk

**Keywords:** whirling disease, proliferative kidney disease, transcriptome, gene ontology, biological pathways

## Abstract

Parasitic infections, such as those caused by the myxozoans *Myxobolus cerebralis* and *Tetracapsuloides bryosalmonae*, pose major threats to wild and farmed salmonids due to severe tissue damage and impairment of the host immune system. While individual infections have been studied, limited information is available on the host response during co-infection. This study investigated the transcriptomic immune response of rainbow trout (*Oncorhynchus mykiss*) during single and sequential co-infections with *M. cerebralis* and *T. bryosalmonae* using RNA-seq. Trout were exposed to single infections (Mc or Tb) followed by co-infections (Mc^+^ or Tb^+^). Fish were sampled at 31 days post-single infection (1 day post-co-infection). RNA from gill and caudal fin (portal of parasite entry) was sequenced, followed by differentially expressed genes (DEGs) identification and GO and KEGG enrichment. In the caudal fin, Mc^+^ (1 day after co-infection with *T. bryosalomne*) fish showed mild immune activation with *C4B* upregulation, while Tb^+^ fish exhibited a stronger response involving *IFI44, ISG15, RSAD2, and TLR7* signaling. In gills, Mc^+^ fish showed moderate cytokine-related gene upregulation, while Tb^+^ (1 day after co-infection with *M. cerebralis*) fish displayed increased expression of humoral response genes (*C3*, immunoglobulin pathways) but suppression of genes involved in B cell development. These results indicate that the order of infection shapes the outcome of the host immune response, offering candidate targets at the host–pathogen interface.

## 1. Introduction

Salmonid populations in both wild and aquaculture environments are impacted by two major myxozoan diseases, whirling disease (WD) and proliferative kidney disease (PKD), which are caused by *Myxobolus cerebralis* and *Tetracapsuloides bryosalmonae*, respectively. Both diseases are associated with substantial economic impacts on the aquaculture industry [1] across diverse geographic regions, most notably in North America and Europe [1,2,3,4]. WD is a serious disease in salmonid fish that severely compromises host function and mobility [5]. Rainbow trout can exhibit infection rates approaching 100%, with high mortality and pronounced clinical signs, depending on the intensity of exposure to parasitic spores [6]. *M. cerebralis* possesses a two-host life cycle that requires both a salmonid fish and the oligochaete worm *Tubifex tubifex*. The worm releases triactinomyxon spores (TAMs), which infect salmonid hosts through the skin, gill epithelium, or oral route [7]. In the initial host compartments, the epidermis and dermis, some parasites undergo spontaneous degeneration, potentially due to a humoral immune response within the fish skin. These early interactions at the skin level may play a critical role in determining the varying degrees of resistance observed among different salmonid species [8,9].

PKD is a temperature-dependent disease affecting both cultured and wild salmonids. It primarily occurs at water temperatures above 15 °C during the summer and fall. Infection rates can reach up to 100% of the stock, with mortalities as high as 95% [10]. The life cycle of *T. bryosalmonae* involves two hosts: a vertebrate salmonid fish and an invertebrate freshwater bryozoan (*Fredricella sultana*). Infective spores are released from bryozoans into the water and enter the fish host via the gill epithelium or skin [11,12]. Upon attachment, the parasite invades epidermal cells and disseminates through the vascular system, ultimately localizing in the kidney—the primary target organ—where clinical signs typically develop within a few weeks [13].

In concomitant infections, multiple infectious agents are present simultaneously within a single host [14]. Co-infections can have significant adverse effects on the host, including altered susceptibility and changes in the course and duration of infection [15]. Interactions between coexisting pathogens during co-infection may be either synergistic or antagonistic, further influencing disease outcomes [14,15,16]. A study on mixed infection with *Kudoa islandica* and *Nucleospora cyclopteri* reported an increased mortality rate [17]. Similarly, a report on co-infection with sympatric myxozoans in salmonids has demonstrated varying degrees of inter-parasite competition within the host [15]. Primary infection with *T. bryosalmonae* was found to have a greater influence on the development of *Chloromyxum schurovi* [18,19]. The co-existence of *T. bryosalmonae* and *M. cerebralis* in rainbow trout has also been investigated. When *M. cerebralis* infection occurred first, followed by *T. bryosalmonae*, synergistic effects were observed, resulting in more severe impacts on the host. Conversely, in cases where *T. bryosalmonae* infection preceded *M. cerebralis*, clinical signs of WD were reduced, although the mortality rate remained unchanged [5]. In a co-infection study involving WD and PKD, Kotob et al. investigated the immune response of rainbow trout following sequential exposure to *M. cerebralis* and *T. bryosalmonae* [5]. They reported that co-infection, particularly when *M. cerebralis* preceded *T. bryosalmonae*, led to a synergistic increase in parasite burden and a marked upregulation of *SOCS-1* and *SOCS-3*. These changes suggest a suppression of the JAK/STAT signaling pathway, contributing to immune suppression and increased disease severity. A recent proteomic study on co-infection reported that 16 proteins were differentially regulated in the caudal fins following *M. cerebralis* exposure, while 27 proteins were modulated in the gills after *T. bryosalmonae* infection. Co-infections altered four proteins in the caudal fins and 11 in the gills, including four myxozoan proteins predicted to function as virulence factors [20].

Physical components such as the mucus layer, scales, and epithelium in the gills and skin are essential elements of the innate immune system and play a critical role in defending against pathogen invasion [21]. To date, no transcriptomic studies have examined host gene modulation at the parasite entry sites during co-infection with PKD and WD. In this study, we performed a transcriptome analysis to identify significant host gene modulation associated with single and co-infections with *T. bryosalmonae* and *M. cerebralis* at the primary portals of entry, caudal fins and gills.

## 2. Results

### 2.1. Differential Expression Analysis in Caudal Fins

**Figure 1:**
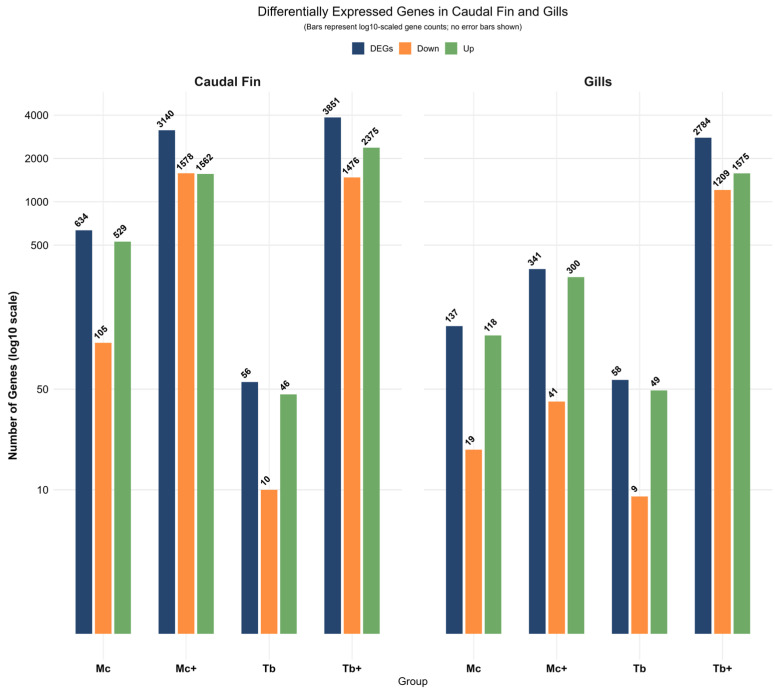
Differentially expressed genes in caudal fin (CF) and gills. DEG, differentially expressed genes.

A total of approximately 1.12 billion high-quality paired-end reads were retained after trimming across all 15 CF RNA-seq libraries, with average read lengths ranging from 114.98 to 127.91 base pairs. Post-trimming, each library contributed between 60.5 and 98.4 million reads (Supplementary File S1). Transcript expression analysis of single-infected and co-infected rainbow trout groups revealed differentially expressed genes (DEGs) in CF (log_2_ fold change > 1; FDR *p*-value ≤ 0.01). Differential expression analysis comparing each infected group (Mc, Mc^+^, Tb, and Tb^+^) to their respective non-infected control tissues revealed 634, 3140, 56, and 3851 DEGs, respectively (Figure 1) (Supplementary File S2).

### 2.2. Differential Expression Analysis in Gills

A total of approximately 818.8 million clean paired-end reads were retained after quality trimming across all 15 gills RNA-seq libraries, with average read lengths ranging from 115.08 to 120.57 bases. Each library contributed between 48.1 and 62.8 million reads after trimming (Supplementary File S1). Transcript expression analysis of single-infected and co-infected rainbow trout groups revealed differentially expressed genes (DEGs) in gills (log_2_ fold change > 1; FDR *p*-value ≤ 0.01). Differential expression analysis comparing each infected group (Mc, Mc^+^, Tb, and Tb^+^) to their respective non-infected control tissues revealed 137, 341, 58, and 2784 DEGs, respectively (Figure 1) (Supplementary File S3).

**Figure 2:**
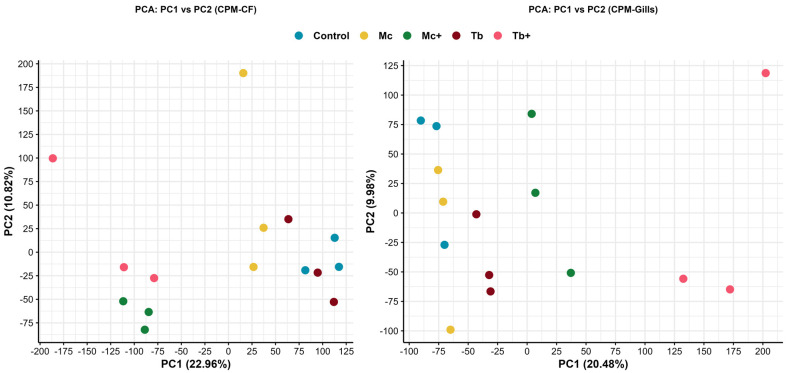
Principal component analysis (PCA) of gene expression profiles in caudal fin (**Left**) and gills (**Right**) based on counts per million (CPM) values.

Principal component analysis based on counts per million (CPM) normalization demonstrated distinct transcriptional profiles among infection groups in both caudal fin and gill tissues. In CF, the first two principal components (PC1 and PC2) accounted for 22.96% and 18.82% of the total variance, respectively. Samples from Mc^+^ and Tb^+^ groups clustered separately from the control group, indicating pronounced transcriptional deviation during parasite-dominant co-infections. Fish infected with *T. bryosalmonae* (Tb) did not exhibit distinct clustering from control fish. In contrast, PCA revealed relatively distinct segregation of *M. cerebralis*-infected (Mc) samples from controls, indicating more pronounced transcriptional changes in the caudal fin, a primary entry route for *M. cerebralis* via the skin. In gill tissues, PC1 and PC2 explained 20.48% and 9.93% of the variance, respectively. While *M. cerebralis*-infected samples (Mc) did not exhibit clear segregation from non-infected (control) samples, PCA revealed moderate or distinct clustering of the Tb, Mc^+^, and Tb^+^ groups compared to controls. The results highlight tissue- and infection-specific differential gene expression patterns (Figure 2).

**Figure 3:**
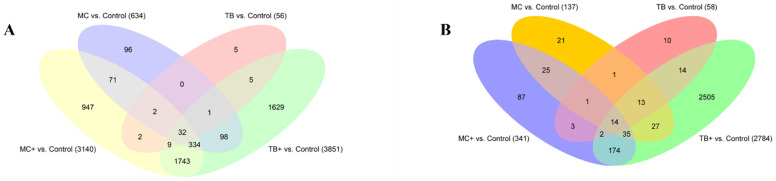
Venn diagram showing the identified genes in the caudal fins (**A**) and gills (**B**) across all groups.

The Venn diagram reveals the distribution of differentially expressed genes in caudal fins across Mc, Mc^+^, Tb, and Tb^+^ groups. Mc^+^ and Tb^+^ exhibit the highest numbers of unique genes, while 32 genes are commonly upregulated in all four groups. Notably, Mc and Tb share no common differentially expressed genes (Figure 3A).

The Venn diagram reveals the differential gene expression in gills across the four groups: Mc, Mc^+^, Tb, and Tb^+^. The Tb^+^ group exhibits the highest number of unique genes (2505), while 14 genes are shared across all groups. Several overlapping subsets are observed, notably between Mc^+^ and Tb^+^, and Mc and Mc^+^ (Figure 3B).

### 2.3. Upregulated Genes in the Caudal Fins (CF)

#### 2.3.1. Upregulated Genes After *M. cerebralis* Infection (Mc)

In response to *M. cerebralis* infection, rainbow trout exhibit a coordinated activation of innate immune pathways alongside cellular restructuring and cytokine signaling activation. Among the 529 upregulated genes, prominent genes such as complement *C4B* Chido blood group (*C4B*), ceruloplasmin (*CP*), interferon induced protein 44 (*IFI44*), N-ethylmaleimide sensitive factor vesicle fusing ATPase (*NSF*), and tetraspanin 7 (*TSPAN7*) suggest a collective strong pro-inflammatory and vesicle trafficking-derived immune activation.

**Figure 4:**
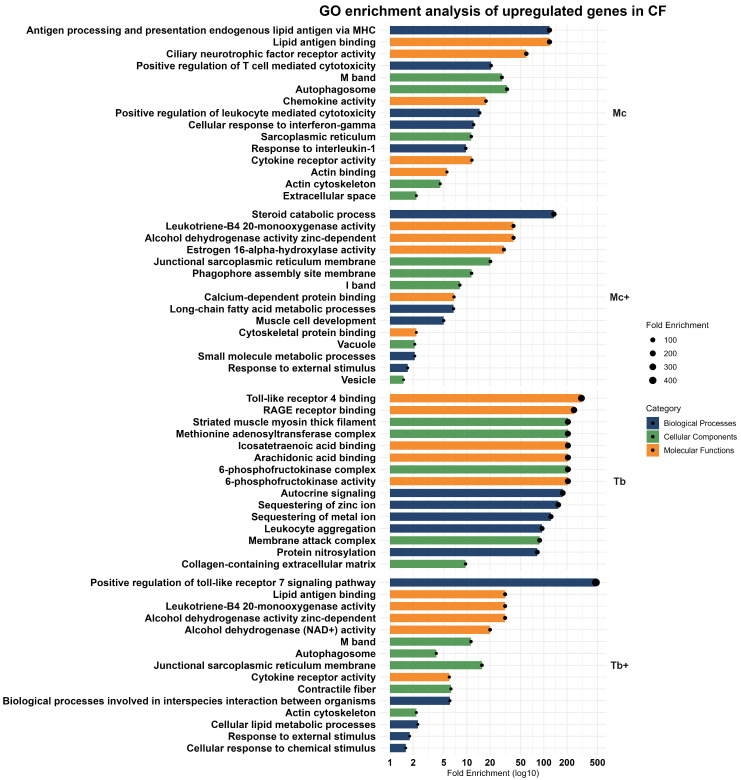
GO enrichment analysis of upregulated genes in caudal fin.

In functional enrichment analysis based on biological processes, key immune processes such as antigen processing and presentation of endogenous lipid antigen via MHC, positive regulation of T cell-mediated cytotoxicity, positive regulation of leukocyte-mediated toxicity, cellular response to interferon-gamma, and response to interleukin-1 were observed. Moreover, the involvement of cellular components such as the autophagosome, sarcoplasmic reticulum, actin cytoskeleton, and extracellular space highlights the structural reorganization of cells during immune engagement and stress response. Upregulated molecular functions like cytokine and chemokine receptor activity, lipid antigen binding, and actin binding suggest an immune strategy that integrates signaling, cell trafficking, and barrier defense mechanisms (Figure 4).

#### 2.3.2. Upregulated Genes After Co-Infection with *T. bryosalmonae* (Mc^+^)

Co-infection with *T. bryosalmonae* infection triggered a broad transcriptional response in rainbow trout, marked by upregulation of 1562 genes including cytochrome P450 family 24 subfamily A member 1 (*CYP24A1*), F-box protein 32 (*FBXO32*), complement *C4B* chido blood group (*C4B*), chromosome X open reading frame 65 (*CXorf65*), interferon induced protein 44 (*IFI44*), and pyruvate dehydrogenase kinase 2 (*PDK2*), associated with immune signaling, metabolic regulation, and cellular stress adaptation. GO enrichment analysis revealed biological processes linked with lipid metabolism (steroid and long-chain fatty acid catabolism), muscle cell development, small molecule metabolic process, and response to external stimuli. The involvement of cellular components such as the sarcoplasmic reticulum membrane, phagophore assembly site membrane, I band, vacuole, and vesicle reflects intracellular remodeling. Enrichment in alcohol dehydrogenase activity, leukotriene-B4 20-monooxygenase activity, estrogen 16-alpha-hydroxylase activity, calcium-dependent protein binding, and cytoskeletal protein binding indicates a coordinated immune–metabolic response (Figure 4).

#### 2.3.3. Upregulated Genes After Infection with *T. bryosalmonae* (Tb)

In *T. bryosalmonae*-infected fish, a moderate but targeted transcriptional response was observed, with 46 significantly upregulated genes linked to interferon signaling, immune modulation, and cellular stress responses. Key upregulated genes included interferon induced protein 44-like (*IFFI44L*), interferon induced protein 44 (*IFI44*), kelch domain containing 1 (*KLHDC1*), guanylate binding protein 1 (*GBP1*), and multivesicular body subunit 12A (*MVB12A*).

Gene ontology analysis revealed contribution of upregulated genes in various biological processes including autocrine signaling, sequestration of zinc ions, sequestration of metal ions, leukocyte aggregation, and protein nitrosylation. These pathways all point towards an immune strategy that combines localized inflammation with metabolic regulation. The cellular component pathways like striated muscle myosin thick filament, the 6-phosphofructokinase complex, the methionine adenosyltransferase complex, the membrane attack complex, and the collagen-containing extracellular matrix indicate coordination between metabolism and tissue structure stabilization. At the molecular level, upregulated genes were involved in the binding of toll-like receptor 4, RAGE receptor, icosatetraenoic acid, arachidonic acid, and 6-phosphofructokinase activity, underscoring an integration of innate immune recognition and glycolytic regulation (Figure 4).

#### 2.3.4. Upregulated Genes After Co-Infection with *M. cerebralis* (Tb^+^)

Co-infection with *M. cerebralis* (Tb^+^) triggered a robust transcriptional response in rainbow trout, leading to the upregulation of 2375 genes, including interferon induced protein 44-like (*IFI44L*), interferon induced protein 44 (*IFI44*), radical S-adenosyl methionine domain containing 2 (*RSAD2*), ISG15 ubiquitin-like modifier (*ISG15*), and receptor transporter protein 5 (Putative) (*RTP5*).

GO analysis revealed enrichment in biological processes such as the positive regulation of the toll-like receptor 7 signaling pathway, interspecies interaction between organisms, cellular lipid metabolic process, response to external stimuli, and cellular response to chemical stimuli. These findings reflect broad spectrum immune and metabolic adaptation to concomitant parasitic infection. Upregulated genes were associated with structural and immune-related cellular components, consisting of junctional sarcoplasmic reticulum membrane, M band, contractile fiber, autophagosome, and actin cytoskeleton, indicating cytoskeletal remodeling and intracellular trafficking. At the molecular level, increased zinc-dependent alcohol dehydrogenase activity, lipid antigen binding, leukotriene-B4 20-monooxygenase activity, alcohol dehydrogenase (NAD+) activity, and cytokine receptor activity suggest immune signaling and lipid metabolism (Figure 4).

### 2.4. Downregulated Genes in the Caudal Fins (CF)

#### 2.4.1. Downregulated Genes in *M. cerebralis*-Infected Group (Mc)

The downregulated transcriptomic profile in *M. cerebralis*-infected caudal fin reflects suppressed cell cycle activity, transcriptional regulation, and protein transport. Key suppressed genes include early growth response 4 (*EGR4*), KLF transcription factor 4 (*KLF4*), prostaglandin-endoperoxide synthase 2 (*PTGS2*), StAR-related lipid transfer domain-containing 4 (*STARD4*), and immediate-early response 2 (*IER2*), suggesting reduced proliferation and stress-adaptive responses.

**Figure 5:**
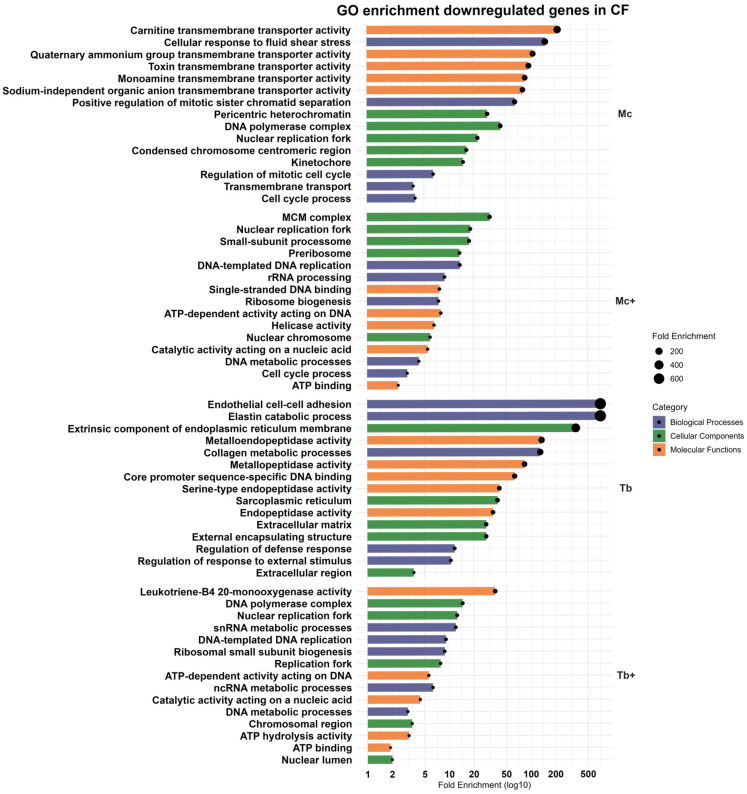
GO enrichment analysis of downregulated genes in caudal fin.

GO enrichment highlighted downregulation of cellular response to fluid shear stress, positive regulation of mitotic sister chromatid separation, regulation of the mitotic cell cycle, cell cycle processes, and transmembrane transport. The cellular components analysis of downregulated genes included DNA polymerase complex, pericentric heterochromatin, nuclear replication fork, condensed chromosome centromeric region, and kinetochore. The most enriched molecular function of downregulated genes included carnitine transmembrane transporter activity, quaternary ammonium group transmembrane transporter activity, toxin transmembrane transporter activity, monoamine transmembrane transporter activity, and sodium-independent organic anion transmembrane transporter activity (Figure 5). Together, these pathways are involved in suppression of proliferative pathways, transmembrane transport, and metabolic regulation.

#### 2.4.2. Downregulated Genes After Co-Infection with *T. bryosalmonae* (Mc^+^)

In total, 1578 genes expressed negative regulation after co-infection, including armadillo-like helical domain containing 4 (*ARMH4*), NME/NM23 nucleoside diphosphate kinase 4 (*NME4*), FOS-like 2, AP-1 transcription factor subunit (*FOSL2*), calcium voltage-gated channel subunit alpha1 A (*CACNA1A*), and unc-45 myosin chaperone B (*UNC45B*).

The downregulated genes showed enrichment for DNA-templated DNA replication, rRNA processing, ribosome biogenesis, DNA metabolic process, and cell cycle process. Suppressed components included the MCM complex, small subunit processome, nuclear replication fork, pre-ribosome, and nuclear chromosome. Molecular functions such as ATP-dependent activity acting on DNA, single-stranded DNA binding, helicase activity, catalytic activity acting on nucleic acid, and ATP binding were also reduced (Figure 5). Collectively, co-infection induced broad suppression of genes related to DNA replication, ribosome biogenesis, and cell cycle progression in the caudal fin.

#### 2.4.3. Downregulated Genes in *T. bryosalmonae*-Infected Group (Tb)

In the *T. bryosalmonae*-infected group, ten genes were downregulated, including ADAM metallopeptidase with thrombospondin type 1 motif 19 (*ADAMTS19*), matrix metallopeptidase 27 (*MMP27*), matrix metallopeptidase 3 (*MMP3*), immediate early response 2 (*IER2*), and thrombospondin (*THBS4*). These genes are involved in extracellular matrix remodeling, with GO enrichment revealing the decreased expression of biological processes including elastin catabolic process, endothelial cell–cell adhesion, collagen metabolic process, regulation of defense response, and regulation of response to external stimuli. The downregulated cellular components consisted of extrinsic components of endoplasmic reticulum membrane, extracellular matrix, external encapsulating structure, extracellular region, and sarcoplasmic reticulum. The molecular functions enriched among these genes were involved in metalloendopeptidase activity, metallopeptidase activity, core promoter sequence-specific DNA binding, serine-type endopeptidase activity, and serine-type endopeptidase activity (Figure 5), indicating a dampened proteolytic and tissue remodeling response.

#### 2.4.4. Downregulated Genes After Co-Infection with *M. cerebralis* (Tb^+^)

In the Tb^+^ (co-infected) group, 1476 genes were downregulated, including ELOVL fatty acid elongase 3 (*ELOVL3*), tetratricopeptide repeat domain 24 (*TTC24*), C2 calcium dependent domain containing 4C (*C2CD4C*), heat shock protein family A (*Hsp70*) member 2 (*HSPA2*), FOS-like 2, and AP-1 transcription factor subunit (*FOSL2*).

These genes are associated with snRNA metabolic process, DNA-templated DNA replication, ribosomal small subunit biogenesis, ncRNA metabolic process, and DNA metabolic process, suggesting the suppression of core transcriptional and translational processes. The cellular components included DNA polymerase complex, nuclear replication fork, chromosomal region, and nuclear lumen. Enriched molecular functions indicated reduced leukotriene B4 20-monooxygenase activity, ATP-dependent activity acting on DNA, catalytic activity acting on nucleic acids, ATP hydrolysis activity, and ATP binding (Figure 5). A comprehensive overview of the enriched GO terms in the CF and associated gene counts are presented in Table 1. Further information regarding enriched pathways in CF is provided in Supplementary File S4.

### 2.5. KEGG Pathway Analysis of Caudal Fin DEGs

**Figure 6:**
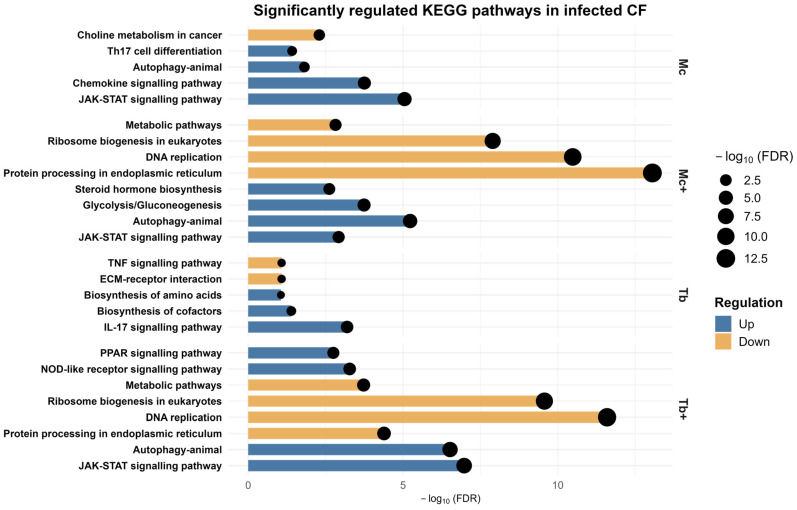
KEGG pathway enrichment analysis in caudal fin.

Pathways related to immune signaling, metabolic processes, and cellular stress were identified using the Kyoto Encyclopedia of Genes and Genomes (KEGG) pathway database (Supplementary File S5). Notably, JAK-STAT and autophagy pathways were commonly enriched in Mc, Mc^+^, and Tb^+^ groups. The upregulated differentially expressed genes (DEGs) in the Mc fish revealed the activation of key immune signaling cascades, including the chemokine signaling pathway, and Th17 cell differentiation. This suggests an early pro-inflammatory and immune regulatory response. The downregulation of metabolism-related pathways involved choline metabolism. Co-infection with *T. bryosalmonae* (Mc^+^) intensified this response, with enriched pathways for glycolysis, gluconeogenesis, and steroid hormone biosynthesis. Downregulated pathways in Mc^+^ also included DNA replication, ribosome biogenesis, and protein processing in the endoplasmic reticulum, indicating a shift from cellular proliferation towards defense. In fish infected with *T. bryosalmonae* (Tb), a more focused immune–metabolic response was evident. Upregulated genes enriched IL-17 signaling, amino acid biosynthesis, and cofactor production, reflecting immune activation with metabolic adjustments, while downregulated genes were enriched with ECM-receptor interaction and TNF signaling pathways. The Tb^+^ co-infected group showed the most complex transcriptomic signature, with distinctly enriched PPAR and NOD-like receptor signaling indicating a robust and multifaceted response combining innate immunity, lipid metabolism, and pathogen recognition. Simultaneously, suppression of DNA replication, ribosome biogenesis, ER protein processing, and broad metabolic pathways point to a cellular stress state (Figure 6). The number of genes associated with each enriched KEGG pathway in the CF are shown in Table 2.

### 2.6. Upregulated Genes in Gills

#### 2.6.1. Upregulated Genes in the Gills After a Single Infection with *M. cerebralis* (Mc)

**Figure 7:**
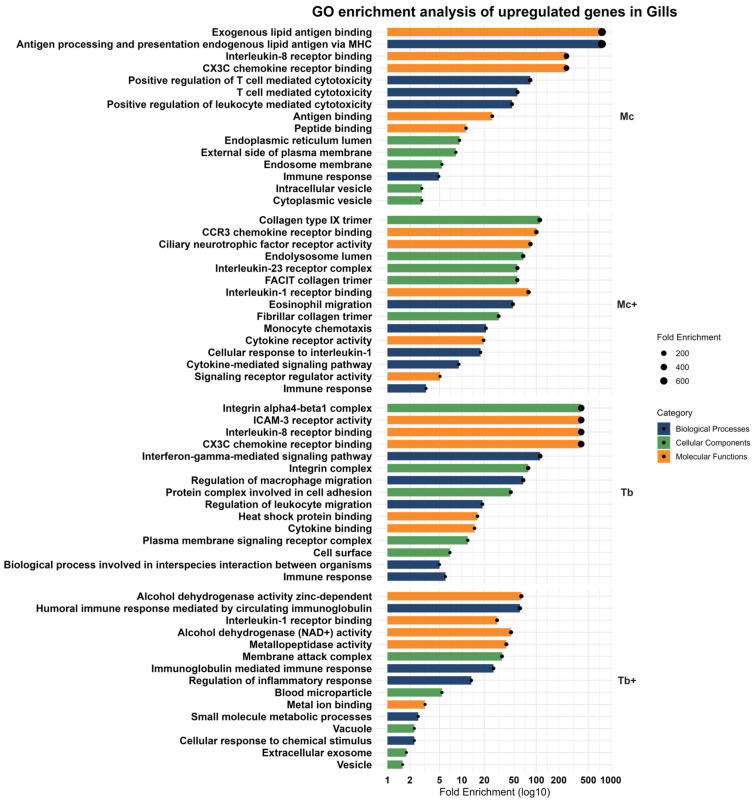
GO enrichment analysis of upregulated genes in Gills.

A total of 118 upregulated DEGs were observed in this group, which consists of overly expressed genes like chitinase acidic (*CHIA*), afamin (*AFM*), serpin family A member 4 (*SERPINA4*), apolipoprotein A1 (*APOA1*), and interferon-induced protein 44-like (*IFI44L*). These genes were primarily involved in biological processes associated with antigen processing and presentation of endogenous lipid antigens via MHC, as well as positive regulation of T cell-mediated cytotoxicity, leukocyte-mediated cytotoxicity, T cell-mediated immunity, and immune response, reflecting a localized activation of adaptive and innate immune response at the mucosal surface. Enriched cellular components pathways such as the blood microparticle, endosome membrane, endosome, cytoplasmic vesicle, and intracellular vesicle supports intracellular trafficking and secretion of immune mediators. The associated molecular functions affected included exogenous lipid antigen binding, interleukin-8 receptor binding, CX3C chemokine receptor binding, antigen binding, and peptide binding (Figure 7).

#### 2.6.2. Upregulated Genes in the Gills After Co-Infection with *T. bryosalmonae* (Mc^+^)

In response to co-infection with *T. bryosalmonae* (Mc^+^), an intensified response with 300 genes mainly involved in immune response was observed. Upregulated genes include poly(A) binding protein cytoplasmic 1 like (*PABPC1L*), complement *C4B* (Chido blood group) (*C4B*), TNF receptor superfamily member 6b (*TNFRSF6B*), desert hedgehog signaling molecule (*DHH*), interferon-induced protein 44 (*IFI44*), and F-box protein 32 (*FBXO32*). These genes were associated with eosinophilic migration, monocyte chemotaxis, cellular response to interleukin-1, cytokine-mediated signaling pathways, and immune responses, probably marking a shift toward inflammatory cell recruitment. Cellular components such as collagen type IX trimer, endolysosome lumen, FACIT collagen trimer, interleukin-23 receptor complex, and fibrillar collagen trimer point to active-matrix remodeling and cytokine response. Molecular enrichment included CCR3 chemokine receptor binding, ciliary neurotrophic factor receptor activity, interleukin-1 receptor binding, cytokine receptor activity, and signaling receptor regulator activity, reflecting a regulatory interplay between immune and epithelial tissues (Figure 7).

#### 2.6.3. Upregulated Genes in the Gills After Single Infection with *T. bryosalmonae* (Tb)

In the *T. bryosalmonae* single-infected group (Tb), a modest response was observed, with 49 upregulated genes such as interferon induced protein 44-like (*IFI44L*), cerebellin 3 precursor (*CBLN3*), receptor transporter protein 5 (putative) (*RTP5*), eomesodermin (*EOMES*), and guanylate binding protein 1 (*GBP1*). Enrichment analysis of these genes revealed involvement in biological processes such as the interferon-gamma-mediated signaling pathway, regulation of microphage migration, leukocyte migration, immune response, and biological processes involved in interspecies interactions between organisms playing an immune surveillance role. The most enriched cellular components were associated with the integrin alpha4-beta1 complex, a protein complex involved in cell adhesion, a plasma membrane signaling receptor complex, and the cell surface. Functionally, genes exhibited interleukin-8 receptor binding, ICAM-3 receptor activity, CX3C chemokine receptor binding, heat shock protein binding, and cytokine binding (Figure 7).

#### 2.6.4. Upregulated Genes in the Gills After Co-Infection with *M. cerebralis* (Tb^+^)

The strongest transcriptional activation occurred after coinfection with *M. cerebralis* (Tb^+^), with 1575 upregulated genes including complement C3 (*C3*), C-reactive protein (*CRP*), cerebellin 3 precursor (*CBLN3*), tryptophan 2,3-dioxygenase (*TDO2*), interferon-induced protein 44 (*IFI44*), and afamin (*AFM*).

GO enrichment revealed robust induction of humoral immune response mediated by circulating immunoglobulin, immunoglobulin-mediated immune response, regulation of inflammatory response, small molecule metabolic process, and cellular response to chemical stimulus. Cellular localization to the membrane attack complex, blood microparticles, vacuoles, extracellular exosomes, and vesicles suggests amplified vesicle-mediated immune effector deployment. Molecular functions highlighted alcohol dehydrogenase activity (zinc-dependent), metallopeptidase activity, metal ion binding, alcohol dehydrogenase (NAD+) activity, and interleukin-1 receptor binding pathways (Figure 7).

### 2.7. Downregulated Genes in the Gills

#### 2.7.1. Downregulated Genes in the Gills After *M. cerebralis* Infection (Mc)

Out of 1278 downregulated genes, 19 were identified in this group, which contained 2 of the most downregulated annotated genes, unc-13 homolog C (*UNC13C*) and TSC22 domain family member 3 (*TSC22D3*).

**Figure 8:**
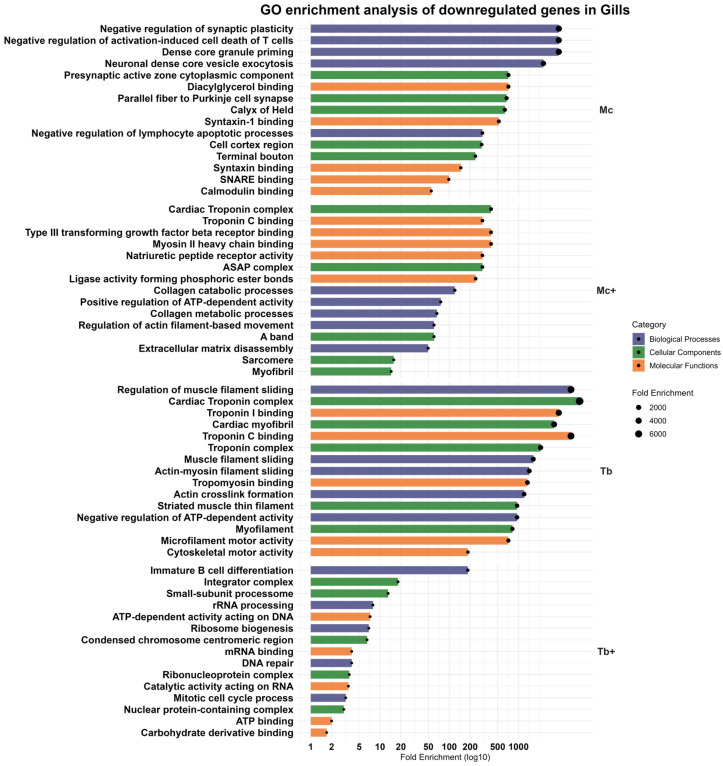
GO enrichment analysis of downregulated genes in gills.

Enriched pathways reflected inhibition of synaptic plasticity, vesicle priming, and T cell apoptotic regulation, with affected cellular components including the presynaptic active zone and Purkinje cell synapse. The molecular functions potentially affected included diacylglycerol binding, syntaxin-1 binding, syntaxin binding, SNARE binding, and calmodulin binding (Figure 8). SNARE binding and calmodulin interaction suggest decreased neurosensory signaling and immune modulatory vesicle trafficking at mucosal surfaces.

#### 2.7.2. Downregulated Genes in the Gills After Co-Infection with *T. bryosalmonae* (Mc^+^)

Co-infection with *T. bryosalmonae* (Mc^+^) led to more pronounced suppression of muscle- and matrix-associated genes such as myosin light chain 3 (*MYL3*), troponin T2, cardiac type (*TNNT2*), natriuretic peptide receptor 2 (*NPR2*), matrix metallopeptidase 27 (*MMP27*), and RNA 2′,3′-cyclic phosphate, and 5′-OH ligase (*RTCB*). Biological processes like collagen catabolic process, positive regulation of ATP-dependent activity, collagen metabolic process, regulation of actin filament-based movement, and extracellular matrix disassembly were enriched. Cellular components like cardiac troponin complex, ASAP complex, A band, sarcomere, and myofibril enrichment predicted impaired structural maintenance and vascular regulation. The most enriched molecular functions were related to myosin II heavy chain binding, type III transforming growth factor beta receptor binding, natriuretic peptide receptor activity, troponin C binding, and ligase activity forming phosphoric ester bonds (Figure 8).

#### 2.7.3. Downregulated Genes in the Gills Following Single Infection with *T. bryosalmonae* (Tb)

In *T. bryosalmonae*-only-infected gills (Tb), downregulation was minimal, but the notable suppression of *TNNT2* reflected targeted interference with contractile fiber dynamics. The biological processes involved the regulation of muscle filament sliding, actin-myosin filament sliding, actin crosslink formation, and negative regulation of ATP-dependent activity. Cellular components’ enriched pathways were associated with troponin complex, striated muscle thin filament, and myofilament. Molecular functions with enriched downregulated pathways included troponin C binding, troponin I binding, tropomyosin binding, microfilament motor activity, and cytoskeletal motor activity (Figure 8). Major biological processes and molecular function-related terms point to modest cytoskeletal impairment.

#### 2.7.4. Downregulated Genes in the Gills Following Co-Infection with *M. cerebralis* (Tb^+^)

The Tb^+^ co-infected group exhibited the most extensive transcriptional suppression, with over 1200 downregulated genes, including calcium voltage-gated channel subunit alpha1 A (*CACNA1A*), serine protease 16 (*PRSS16*), recombination activating 1 (*RAG1*), syndecan binding protein (*SDCBP*), and solute carrier family 16 member 3 (*SLC16A3*).

Downregulated processes included immature B cell differentiation, rRNA processing, ribosome biogenesis, DNA repair, and mitotic cell cycle process. Enriched cellular components included the integrator complex, small subunit processome, condensed chromosome centromeric region, ribonucleoprotein complex, and nuclear protein-containing complex with decreased molecular functions such as ATP-dependent activity acting on DNA, mRNA binding, catalytic activity acting on RNA, ATP binding, and carbohydrate derivative binding (Figure 8). All enriched pathways identified in the gills, along with the corresponding gene counts, are summarized in Table 3. Supplementary File S4 provides detailed information on the identified enriched pathways in gills.

### 2.8. KEGG Pathway Analysis of Gills DEGs

**Figure 9:**
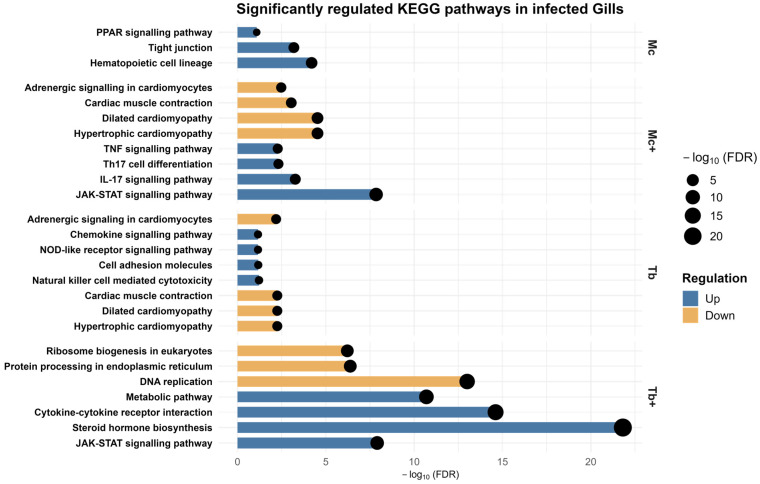
KEGG pathway enrichment analysis in gills.

KEGG pathway analysis of gill DEGs revealed distinct immune-, metabolic-, and stress-associated responses across infection groups (Supplementary File S5). In *M. cerebralis*–infected fish (Mc), upregulated genes were linked to barrier maintenance and metabolic-related pathways such as hematopoietic cell lineage, tight junction, and PPAR signaling pathways. In Mc^+^ fish, pathway enrichment included *IL-17*, TNF signaling, and Th17 cell differentiation. In Tb fish, KEGG-related upregulated pathways were associated with natural killer-cell-mediated cytotoxicity, cell adhesion molecules, NOD-like receptor signaling pathway, and chemokine signaling pathway. In Tb^+^ fish, the enriched pathways were steroid hormone biosynthesis, cytokine–cytokine receptor interaction, and metabolic pathways. Notably, JAK-STAT signaling was enriched as a common upregulated pathway in Mc^+^ and Tb^+^ fish. No significantly enriched KEGG pathways were identified in the Mc group based on the applied threshold criteria. Downregulated genes in Tb and Mc^+^ fish were associated with cardiac muscle contraction, hypertrophic cardiomyopathy, dilated cardiomyopathy, and adrenergic signaling in cardiomyocytes. Suppressed pathways in the Tb^+^ group involved DNA replication, ribosome biogenesis in eukaryotes, and protein processing in the endoplasmic reticulum, broadly limiting the cellular biosynthesis (Figure 9). Table 4 shows the enriched KEGG pathways in the gills along with the number of identified genes.

## 3. Discussion

Multi-parasitic interactions influence both host and parasite populations and significantly affect infection dynamics [22]. Co-infections can have serious impacts on hosts, including changes in susceptibility, disease progression, and duration of infection [14,15]. Co-infection with whirling disease (WD) and proliferative kidney disease (PKD) has been shown to cause pathological changes, high mortality rates, and proteomic changes in rainbow trout [15].

Due to the valuable insights obtained through next-generation sequencing, RNA-seq has been extensively utilized in various transcriptomic studies on myxozoan parasites [23]. For example, a transcriptome-based study examined the immune response of rainbow trout infected with *T. bryosalmonae* [24]. Similarly, the immunological response in hybrid tilapia infected with *Myxobolus bejeranoi* was studied using RNA-seq [25]. Recently, a comparative transcriptomic analysis of *T. bryosalmonae* was conducted using RNA-sequencing on the diseased kidney tissues of rainbow trout and brown trout [26].

However, the transcriptomic modulation of rainbow trout to single and co-infection with *M. cerebralis* and *T. bryosalmonae* has not yet been explored. In the present study, we report the transcriptomic modulation in rainbow trout following single and co-infection with *M. cerebralis* and *T. bryosalmonae*.

In the current study, samples were collected at day 1 post-co-infection to assess the early immune response to a subsequent myxozoan infection in the context of an established primary infection. At this time point, the primary infection had progressed to an advanced stage, marked by pronounced lymphocytic infiltration and cytokine modulation [27]. This experimental design facilitates the detection of immune priming, interference, or suppression. A previous co-infection study with a similar temporal framework elucidated how the order and timing of pathogen exposure influence host immune regulation and disease progression [5]. This research revealed substantial differences in the number of differentially expressed genes (DEGs) between infection groups and tissues, reflecting the varying magnitude of host transcriptional responses. In CF, the Tb^+^ group exhibited the strongest response with 3851 DEGs (2375 upregulated, 1476 downregulated), followed by Mc^+^ (3140 DEGs), Mc (634 DEGs), and Tb (56 DEGs). Likewise, in the gills, the Tb^+^ group had the greatest DEG count (2784), followed by Mc^+^ (341 DEGs), Mc (137 DEGs), and Tb (58 DEGs). These patterns indicate that co-infection, particularly with *T. bryosalmonae* as a primary agent, triggers a robust and complex transcriptional response. The pronounced upregulation of a high number of genes in co-infected groups compared to single-infected fish may reflect elevated immune activation or compensatory mechanisms in response to a subsequent pathogen challenge. The following sections focus mainly on immune-related and metabolic-related genes that exhibited the highest fold changes in response to single and co-infections.

### 3.1. Transcriptomic Response to M. cerebralis Single Infection (Mc)

The complement system is essential to innate immunity and promotes opsonization, inflammation, and lysis of pathogens. The complement component C4 plays a central role in the classical pathway of the complement system [28]. The complement system is known to contribute to the protection of fish against parasitic infections. In salmonids, for example, activation of the alternative complement pathway has been shown to reduce the activity of *Gyrodactylus salaris* and *Discocotyle sagittate* [29]. In rainbow trout, *C3* expression increases in skin mucus and lymphoid tissues upon *Ichthyophthirius multifiliis* infection [30]. Specifically, *C4B* is pivotal in complement activation and the subsequent immune stimulation in rainbow trout [28]. In our study, *C4B* was significantly upregulated following *M. cerebralis* single and co-infection by 55.67-fold and 92.98-fold, respectively, and comparatively to a lesser extent in the gills of Mc^+^ co-infected fish. These observations indicate strong activation of the innate immune system in response to the myxozoan infection. The complement system activation, potentially driven by *C4B* overexpression, may be linked with the severe pathological conditions observed in Mc^+^ fish [5]. Supporting this, Saleh et al. [20] reported an increased abundance of complement factor H as a host immune strategy to restore homeostasis, which reflects homeostatic imbalance and underlines complement overexpression.

Ceruloplasmin (*CP*), an acute-phase protein, limits pathogen growth and contributes to host defense. In addition to its antimicrobial activity, *CP* is involved in antioxidative defense and iron metabolism. Its expression was upregulated in fish infected with *Edwardsiella ictalurid* [31]. In the current study, we observed elevated *CP* expression correlating with *M. cerebralis* infection severity, suggesting a role in inflammation. This observation supports previous findings [32], though further studies are needed to assess its potential as an early biomarker for whirling disease (WD).

Tetraspanins (*TSPANs*) are membrane proteins involved in immune regulation and cell adhesion. In zebrafish, *TSPANs* interact with selectins, laminins, and integrins to mediate immune cell migration. In rainbow trout, immune cells expressing *CD9* and *CD63* play roles in B cell activation and host defense [33] and are implicated in responses to viral hemorrhagic septicemia virus (VHSV) and infectious pancreatic necrosis virus (IPNV) [34,35]. In our study, *TSPAN7* was strongly upregulated (21.38-fold) in *M. cerebralis*-infected fish, potentially reflecting an activated immune state. However, functional studies are needed to clarify the specific role of *TSPANs* during WD.

*NSF*, an ATPase regulating SNARE complex disassembly via α-SNAP and ATP hydrolysis [36], was upregulated, indicating increased vesicle cycling possibly linked to antigen processing and immune signaling. Changes in vesicle trafficking proteins, including clathrin heavy chain, SH3-domain proteins, and N-ethylmaleimide-sensitive factor (*NSF*), suggest active intracellular transport remodeling. These components are central to clathrin-mediated endocytosis (CME), a route often used by pathogens [20]. Further research may clarify how *M. cerebralis* modulates host vesicular pathways.

Gene ontology (GO) analysis of upregulated genes in the caudal fin (CF) revealed enrichment of immune-related, cytoskeletal, and neural signaling pathways. Previous studies reported elevated IgM^+^ B cells, myeloid cells, and proliferating CD8^+^ T cells in the CF of WD affected trout [37]. In our data, enriched processes included antigen presentation via MHC, cytotoxicity, response to *IL-1*, and autophagy consistent with these findings. Lipid-based endogenous antigens are presented via MHC class I to *CD8^+^* T cells, promoting cytotoxic responses [38]. Conversely, downregulation of *MHC II* observed in *Gyrodactylus derjavini*-infected trout suggests parasite-mediated immune evasion [39]. Our findings suggest that *M. cerebralis* activates peripheral innate immunity while overlooking adaptive responses. Upregulated genes also involved chemokine and interleukin pathways, such as chemokine activity, cytokine receptor signaling, and *IL-1* response.

Chemokines bridge innate and adaptive immunity by recruiting leukocytes to infection sites [40]. Their upregulation in our study is consistent with responses reported for *T. bryosalmonae*, *Cryptocaryon irritans*, and *I. multifiliis* [24,41,42]. Cytokine receptor activity was also enriched. Interleukins *IL-2*, *IL-3*, *IL-11*, *IL-12*, and *IL-23* were upregulated in CF, suggesting engagement of *Th1* and *Th17* pathways. *IL-12* promotes *Th1* responses via *IFN-α* and STAT signaling in Nile tilapia [43], while *STAT3* is linked to *Th17*-mediated responses in *M. cerebralis* infection [44].

Ciliary neurotrophic factor (*CNTF*), part of the *IL-6* cytokine family, is expressed in glial cells and supports neural and muscular maintenance [45,46]. The upregulation of *IL13RA1*, *OSMR*, *CRLF2*, and *IL6R* in our study may indicate responses to maintain neuromuscular integrity under infection-induced stress.

*Krüppel-like factor 4* (*KLF4*), a transcription factor essential for cellular differentiation, vascular stability, and immune regulation [47], was downregulated (−4.92-fold), possibly compromising epithelial and immune functions. Similarly, *PTGS2*, involved in leukocyte activation and inflammation [48], was downregulated (−4.75-fold), suggesting suppressed inflammatory signaling.

Downregulated genes were also enriched in pathways related to DNA replication, cell division, and solute transport. Suppressed expression of *SLC22A* family genes, which transport endogenous and xenobiotic compounds [49], may reflect metabolic reprogramming or parasite-driven interference with host homeostasis.

In gills, *SERPINA4* was the most modulated gene. Serpins regulate immune responses by inhibiting proteases involved in inflammation, coagulation, and complement activation [50,51,52]. Previous studies reported elevated *serpin G1* and *A8* in infected mucosal tissues of catfish [53]. Upregulation of *SERPINA4* likely reflects early mucosal immune activation and tissue preservation.

*Apolipoprotein A1* (*ApoA1*)—involved in lipid transport and antibacterial defense—was also upregulated. In both channel catfish and trout, *ApoA1* is expressed in mucosal tissues and supports immune protection [54,55]. Its increased expression in gills suggests a barrier-protective and anti-inflammatory role during *M. cerebralis* infection.

GO terms related to antigen processing and presentation, including MHC-mediated lipid antigen presentation, suggest active antigen-presenting cell (APC) activity in the gills. Upregulation of T cell-mediated and leukocyte-mediated cytotoxicity pathways supports adaptive immune activation. Prior transcriptomic studies in *T. bryosalmonae*-infected trout showed the upregulation of *IFN-γ*, *CD8^+^*, and *IL-2* [27]. In *Carassius auratus langsdorfii*, *CD8^+^* T cells exhibited innate cytotoxicity against *I. multifiliis* via perforin and serine proteases [56]. In fish, adaptive immunity involves V(D)J recombination to generate diverse *TCRs* and *BCRs* [57]. Cytotoxicity is mediated by *TCR^+^CD8^+^* lymphocytes (MHC-restricted) and *NCCRP-1^+^* NK-like cells (MHC-independent) [58]. After exposure to *M. cerebralis*, CD8^+^ T cells showed expansion systemically and locally in WD-resistant rainbow trout [59], likely contributing to parasite control.

The *UNC13* gene family regulates vesicle fusion. *UNC13D* is expressed in endothelial cells and supports lumen formation in zebrafish [59]. In our study, downregulation of *UNC13C* may reflect impaired tissue morphogenesis during infection. *TSC22D3*, a transcription factor with immunosuppressive properties, is downregulated in *Piscirickettsia salmonis*-infected Atlantic salmon, possibly reflecting a shift toward inflammation [60]. Similarly, we observed *TSC22D3* downregulation, suggesting altered T cell regulation and enhanced inflammatory responses in *M. cerebralis*-infected trout.

### 3.2. Transcriptomic Response to Co-Infection with T. bryosalmonae (Mc^+^)

Cytochrome P450 family 24 subfamily A member 1 (*CYP24A1*), a vitamin D receptor target gene, degrades active vitamin D and acts as a negative regulator of its signaling, thereby reducing cell growth and immune modulation [61,62]. Its 145.7-fold upregulation may represent either a host-driven strategy to control cell proliferation or a parasite-mediated mechanism to suppress immunity.

In rainbow trout, F-box protein 32 (*FBXO32*) plays a key role in the degradation of muscle proteins, particularly under conditions of stress, nutritional deficiency, or immune activation. Elevated *FBXO32* levels during infection may contribute to muscle atrophy, potentially serving as a mechanism for energy conservation [62]. Another study reported a positive correlation between stress (induced due to fasting) and the upregulation of *FBXO32* expression in zebrafish [63]. In the present study, the overexpression of this gene (92.98-fold) may reflect stress induced by disease conditions; however, the precise role of this protein in response to infection in fish remains to be elucidated.

Cortisol is known to suppress immune responses, increase parasite load, and elevate mortality in rainbow trout [64]. In Nile tilapia infected with *Myxobolus* spp. and *Henneguya* spp., serum cortisol levels were found to be higher compared to uninfected fish [65]. The marked 35.5-fold upregulation of *PDK2* likely reflects cortisol-induced stress in response to co-infection [66].

Upregulated GO terms in the Mc^+^ group were linked to metabolism, muscle function, autophagy, cellular maintenance, and inflammation regulation. Enrichment of long-chain fatty acid metabolism suggests a shift toward lipid utilization under prolonged stress [66]. The leukotriene-B4 20-monooxygenase pathway, involved in inflammation, was also enriched [67]. Given that gills are the primary entry site for *T. bryosalmonae*, a moderate local immune response at this stage is expected. The absence of protein synthesis and immunogenic pathway upregulation aligns with previous co-infection proteomic data [20], possibly reflecting immune suppression, supporting subsequent infection with *T. bryosalmonae*. Enrichment of omegasome and phagophore assembly site pathways indicates the activation of autophagy, a catabolic process for recycling cellular components [68]; such activity may represent a compensatory response to stress due to infection-induced tissue damage.

In co-infected rainbow trout, downregulated GO terms were associated with DNA replication and repair, ribosome biogenesis, RNA processing, the cell cycle, and ATP-related processes. Parasite dependence on host protein synthesis may contribute to suppressed ribosome biogenesis [23], while host stress responses may further limit these pathways to conserve energy.

Consistent with previous findings in Jinhu grouper infected with viral nervous necrosis virus (VNN) [69], we observed downregulation of *POLA*, *MCM* complex, and *RFC2*, all involved in DNA-templated replication. In *Oncorhynchus masou*, co-infection with IHNV and *Flavobacterium psychrophilum* led to earlier disease onset and higher mortality, accompanied by increased energy demand and activation of fructose and pentose phosphate pathways [70].

Kidney pathology from *T. bryosalmonae* induces inflammation and hypoxia, reducing mitochondrial ATP production [26]. This likely contributes to the observed suppression of ATP-related pathways, initially triggered by *M. cerebralis* and further exacerbated by co-infection. The mechanisms underlying these changes require further investigation.

Gills expressed upregulation of *DHH* in response to co-infection. In fish, gonadal development is the primary function of the desert hedgehog (*DHH*) signaling molecule [71,72]. The hedgehog signaling pathway is also involved in epithelial maintenance and immune regulation in vertebrates. The potential expression of *DHH* in peripheral tissues such as the skin or gills during infection may indicate a role in tissue regeneration or immune modulation [72]. The 12.60-fold upregulation of *DHH* may suggest its role in maintaining epithelial homeostasis or supporting tissue recovery following parasite-induced damage.

TNF receptor superfamily member 6B (*TNFRSF6B*) was significantly upregulated (14.85-fold) in the gills of co-infected trout, consistent with its upregulation in *Salmo salar* during multifactorial gill disease (MGD) [73]. *TNFRSF6B* is linked to JAK-STAT and TNFR2 signaling and may help regulate gill inflammation by inhibiting epithelial apoptosis, promoting tissue repair, and modulating immune responses. This suggests a conserved role in mucosal immunity across teleosts. Additionally, *FBXO32* was also upregulated in the caudal fin of Mc^+^ fish, reflecting similar stress responses and metabolic adjustments in peripheral tissues during co-infection.

Gene ontology analysis of upregulated genes in the Mc^+^ group showed the enrichment of immune-related pathways, including immune cell migration, cytokine signaling, and adaptive immunity [74]. As part of the adaptive response, T cells recognize antigens via MHC molecules [75], and in fish, CD4^+^ T helper cells regulate other immune cells through cytokine secretion [76]. Upregulation of CD4-related genes such as *CX3CL1*, *IL12*, and *CCL26* indicates active T cell differentiation and maturation. *IL-23* promotes *Th17* cell development, which produces *IL-17*, a pro-inflammatory cytokine important in both defense and autoimmunity [77]. Early upregulation of *IL-1β*, *IL-6*, and *IL-8* suggests mucosal immune activation, while *IL-10* helps restrain excessive inflammation. The induction of *IL-17A*, *IL-22*, and *IL-4* reflects enhanced barrier protection and immune regulation, likely representing a coordinated host response to balance tissue integrity and parasite control [78]. Further study of interleukin dynamics in fish may clarify immune regulation during co-infection.

In our co-infection study, *MYL3* and *TNNT2* downregulation indicates the suppression of genes essential for striated muscle contraction. *TNNT2* is key for muscle development and function [79], while *MYL3* modulates contraction speed in skeletal muscle [80]. Their reduced expression may contribute to infection-induced muscular weakness. *TNNT2* was also downregulated in the Tb single-infection group, possibly to reduce swimming performance, suggesting that suppression of this gene may be a general response to *T. bryosalmonae* infection. Supporting this, studies have linked *MYL3* and *TNNT2* expression to swimming performance and muscle function. *MYL3* shows seasonal variation in goldfish [81] and *TNNT2* influences swimming performance in large yellow croaker [82], reinforcing the functional relevance of these changes in host behavior and physiology.

Natriuretic peptide receptor 2 (*NPR2*) regulates vascular tone and responds to blood volume changes in trout, increasing under volume overload and decreasing in saltwater conditions [83]. Its downregulation in our study suggests reduced cardiovascular adaptability during infection. Matrix metalloproteinases (*MMPs*), such as *MMP-13* in rainbow trout, mediate collagen degradation and support tissue remodeling during growth and repair [84]. Downregulation of *MMP27* in co-infected fish may reflect impaired tissue regeneration and a diminished capacity to repair parasite-induced tissue damage.

### 3.3. Transcriptomic Response to T. bryosalmonae Single Infection (Tb)

Kelch motif-containing proteins regulate innate immunity in Pacific white shrimp. *KLHDC2* enhances humoral immunity and protects against *Vibrio parahaemolyticus* [85]. As *KLHDC1* belongs to the same family and was upregulated in this study, it may similarly contribute to immune modulation in fish. Guanylate-binding proteins (*GBPs*) are *IFN*-inducible GTPases involved in antiviral defense, inflammation, and pathogen resistance [86]. In rainbow trout, *GBP* was shown to be induced by *IFN-γ*, suggesting its role in fish immunity. Its upregulation during *T. bryosalmonae* infection may reflect interaction with other interferon-stimulated genes, such as *Mx* proteins [87].

Autocrine signaling can either activate or inhibit immune cells and cytokine production during infection, thereby modulating host responses to pathogens [88,89]. Its enrichment in our study suggests a proper immune system activation in response to *T. bryosalmonae*. This aligns with previous findings showing higher resistance to proliferative kidney disease (PKD) in rainbow trout compared to brown trout [90]. Additionally, upregulation of genes involved in zinc and metal ion sequestration indicates a nutritional immunity strategy, where the host restricts microbial access to essential metals by tying them to host proteins [91]. This strategy may serve as a defense mechanism against *T. bryosalmonae*.

In vertebrates, including teleosts, *ADAMTS* proteins, zinc-dependent enzymes involved in extracellular matrix remodeling, are crucial for tissue integrity and development. In zebrafish, their expression in embryonic and adult tissues supports roles in neural, muscular, and craniofacial development [92]. The 3.09-fold downregulation of *ADAMTS* in *T. bryosalmonae*-infected trout may reflect parasite-mediated interference with tissue remodeling. *Immediate early response 2* (*IER2*), a highly inducible gene associated with cellular defense and early immune responses [93], was also downregulated. This may indicate suppression of stress-related immune signaling or a host attempt to limit inflammation and keep a proper tissue homeostasis during infection.

Another key observation was the downregulation of serine-type endopeptidase (*SP*), which may reflect a host defense strategy. These enzymes have proteolytic activity and are involved in various pathological processes [94,95]. Similarly, the downregulation of serine/threonine protein phosphatase, previously reported [20], aligns with our findings and may help preserve tissue and cellular integrity during parasitic invasion.

In the Tb group, upregulated gill genes were associated with immune activation, cellular stress responses, and tissue repair. These findings are consistent with recent proteomic studies on *T. bryosalmonae* and *M. cerebralis* infections, which reported elevated levels of proteins involved in oxidative stress response and tissue repair [20]. The upregulation of *integrin α4*, a molecule involved in wound healing, also suggests active tissue repair [96]. The chemokine *C-X3-C*, known for promoting pro-inflammatory responses, was induced following *T. bryosalmonae* infection [97]. These gene expression patterns indicate a controlled immune response with concurrent tissue preservation, consistent with the findings of Kotob et al. [97], who observed limited inflammation despite parasite presence. In contrast, *TNNT2* downregulation may reflect parasite-induced physiological stress.

### 3.4. Transcriptomic Response to Co-Infection with M. cerebralis (Tb^+^)

Among the significantly upregulated ISGs was *IFI44*, a type I *IFN*-responsive gene associated with innate antiviral and antiparasitic defenses [36,98]. It contributes to pathogen recognition, signaling, and leukocyte activation [36,37,38], but also serves as a negative regulator of *IFN* signaling, indicating a regulatory mechanism in immune modulation. Previously, the role of *IFN*-γ has been reported in various studies in response to *M. cerebralis* infection [44,99]. In our study, *IFI44* was consistently upregulated in fish infected with *M. cerebralis* and *T. bryosalmonae* and in their respective co-infected groups across both tissues, the caudal fin and gills. The increased expression of *IFI44* aligns with previous findings reporting the activation of the interferon signaling pathway during WD and steady *IFN*-γ upregulation in *M. cerebralis*-infected rainbow trout [44]. Enhanced *IFN* activation has been observed in the skin following *M. cerebralis* infection [100] and in the kidney in response to *T. bryosalmonae* [27]. In addition, upregulation of *IFN*-derived pathways is in accordance with Saleh et al. [37], and *IFN*-γ expression was increased within the caudal fin of brown trout after infection with *M. cerebralis* [101]. In the current study, in the Tb^+^ group, *IFI44* was markedly upregulated in the CF (1003-fold) and gills (362-fold) 1 day post-co-infection, likely due to a primary immune priming by *T. bryosalmonae* infection. Kotob et al. [27] have reported that *T. bryosalmonae*, as a primary pathogen, triggers the innate and adaptive immune response to limit a subsequent infection with *M. cerebralis* [5]. In contrast, the Mc^+^ group showed lower *IFI44* expression than the single *M. cerebralis* infection. This suggests that initial infection with *M. cerebralis* may impair the host’s ability to mount an effective interferon response upon a subsequent exposure to *T. bryosalmonae* [5].

*RSAD2* (*viperin*), another *IFN*-stimulated gene, plays a role in antiviral defense [102]. In *Siniperca chuatsi*, *RSAD2* was highly expressed in immune-related tissues, suggesting its involvement in host defense, even though it was not strongly induced by Poly I:C stimulation [103]. *ISG15*, a ubiquitin-like protein and key effector of the type I *IFN* response, was also upregulated [104]. The co-expression of *RSAD2* and *ISG15* during co-infection suggests a coordinated *IFN*-mediated immune response in the caudal fin. While typically associated with antiviral activity, their upregulation here indicates broader roles in immune modulation during parasitic infection, likely triggered by co-infection-induced or cross-activated *IFN* signaling pathways.

*ELOVL3*, a fatty acyl elongase, contributes to lipid metabolism by elongating saturated and monounsaturated fatty acids into very long-chain fatty acids, supporting tissue integrity in organs such as the skin and liver [105,106]. Reduced *ELOVL3* expression may reflect disrupted barrier function or a host strategy to divert lipid resources toward immune defense. *IFIT1*, which contains nine tetratricopeptide repeat (*TPR*) domains, plays a key role in immune signaling and recognition of pathogen-associated molecular patterns (PAMPs) through protein–protein interactions [107]. The observed downregulation of *TPR* domain-containing proteins may indicate parasite-driven immune evasion.

Hsp70 proteins are stress-inducible chaperones that protect cells by preventing protein aggregation and supporting immune cell survival during thermal-, chemical-, or pathogen-induced stress [108]. The downregulation of *HSPA2* following co-infection may reflect parasite-induced immune suppression or a shift in energy allocation. The transcription factor *c-FOS*, part of the AP-1 complex, regulates immune and stress response genes. In crustaceans, *FOS* acts as a negative immune regulator, where its suppression enhances antimicrobial peptide (AMP) production and reduces viral replication [109]. Our observation of *c-FOS* downregulation aligns with these findings, although its role in co-infected trout remains to be clarified.

Pronounced complement activation was also evident in the gills, highlighting distinct pathway responses to the two myxozoan parasites at the portals of entry. In the Mc fish, we observed a marked upregulation of *C4*, a key component in the classical complement pathway, which implies the activation of the host immune system. As previously discussed in CF, this reflects active engagement of complement system pathways in these tissues. The Tb^+^ group also showed a significant upregulation of *C3* in the gills, a central component of all complement pathways, suggesting a broad complement-mediated response at this site [110]. The upregulation of *C3* reflects a localized immune defense specifically tailored to the gills, which serve as a primary barrier tissue after subsequent co-infection with *M. cerebralis*. *CRP* (C-reactive protein), an acute-phase protein that promotes complement activation and immune clearance [111], showed a 10,157-fold upregulation, while *C3* increased 12,306-fold, indicating a robust acute-phase and innate immune response. This increase likely represents *CRP*-mediated opsonization and *C3*-driven complement activation aimed at effective pathogen clearance at the gill mucosa.

In fish, tryptophan 2,3-dioxygenase (*TDO*) regulates tryptophan metabolism via the kynurenine pathway, contributing to oxidative stress control, immune modulation, and homeostasis [112,113,114]. Its upregulation in co-infected trout suggests a host response to manage immune activation and oxidative stress through tryptophan degradation, potentially limiting excessive inflammation following *M. cerebralis* co-infection. Although fish lack afamin [115,116], the Group-specific component (*Gc*) gene may perform a similar function in antioxidant transport and plasma protein regulation [116]. Upregulation of the *Gc* (afamin ortholog) in co-infected fish likely reflects enhanced antioxidant defense to counteract oxidative stress and metabolic demands during dual infection. This aligns with afamin’s known role in vitamin E transport and redox balance in vertebrates.

Enrichment of GO terms related to interleukin-1 receptor binding indicates *IL-1*-mediated signaling. As a key pro-inflammatory cytokine, *IL-1* modulates immune responses by regulating other cytokines [117]. In Tb^+^ co-infected gills, enrichment of interleukin-1 receptor binding and regulation of the inflammatory response indicate activation of *IL-1*-mediated signaling, a key component of mucosal innate immunity [118]. This finding aligns with the proteomic results reported by Saleh et al. [20], showing that myxozoan co-infections influence inflammatory and antigen presentation pathways. Additionally, enrichment of humoral and immunoglobulin-mediated responses suggests local B cell activation and antibody-mediated defense at the gills against myxozoan parasites.

Among the downregulated genes, *CACNA1A* is notable for its role in neurotransmitter release and motor coordination [119]. Its reduced expression in Tb^+^ gills may indicate impaired neurosensory function or stress-related disruption of local neural pathways. *PRSS16*, primarily expressed in thymic epithelial cells, is involved in MHC class II antigen processing and T cell development [120]. *RAG1*, essential for V(D)J recombination, is critical for adaptive immune cell development; its loss in zebrafish results in the deficiency of B and T cells, with only innate immunity remaining [121]. Downregulation of *PRSS16* and *RAG1* suggests mucosal immunosuppression, likely as part of a parasite-driven immune evasion strategy. *SLC16A3*, encoding monocarboxylate transporter 4 (*MCT4*), supports lactate transport and cellular metabolism. In zebrafish, its expression increases during refeeding, reflecting metabolic recovery [63]. Its downregulation here points to the disrupted metabolic homeostasis of gill tissue after the subsequent coinfection with *M. cerebralis*. Collectively, these findings indicate that co-infection impairs neurological, immune, and metabolic functions of the gills, compromising host resilience.

### 3.5. KEGG Pathway Analysis at the Portals of Entry

In the current study, KEGG-enriched pathways associated with the JAK-STAT signaling pathway were identified in caudal fin (CF) tissue following *M. cerebralis* single infection, as well as in both co-infection groups (Tb^+^ and Mc^+^) across both tissues. These findings align with a previous co-infection study [9]. The elevated expression of genes such as *SOCS*, *IL-23*, *IFN-γR1*, *JAK*, and STAT suggests an immune regulatory response mounted by the fish host after myxozoans invasion. Enrichment of the *IL-17* signaling pathway in Mc and Tb in the caudal fin and gill tissues is consistent with previous findings in WD and PKD in rainbow trout [27]. As noted earlier, *IL-17* is a pro-inflammatory cytokine with limited homology to other cytokines and acts synergistically within the broader inflammatory network [122]. *IL-17* plays a key role in inflammation, granuloma formation, and mucosal defense, and is commonly associated with Th17-like immune responses. In fish, several *IL-17* family members—such as *IL-17*A/F2a, *IL-17*C1, *IL-17*C2, and *IL-17*D—have been identified and shown to be responsive during infection [27]. The upregulation of *IL-17* family members observed in our study likely reflects a Th17-mediated immune response [123] against myxozoan parasites at the portals of entry [27].

Enrichment of autophagy-related pathways was observed in both types of co-infection in caudal fin tissue. One of the key genes involved in these pathways was *GABARAPL1*, an autophagy-related protein essential for the formation of autophagosomes, which degrade long-lived proteins and damaged organelles. Previous studies have shown that *GABARAPL1* expression is significantly elevated in rainbow trout during periods of nutritional deficiency or fasting, supporting energy balance and muscle protein turnover [124]. In the previous proteomic study, clathrin-mediated endocytosis (CME) was proposed as a key strategy employed by *M. cerebralis* for host immune evasion and its survival [20]. In addition, CME appears to play a crucial role in *T. bryosalmonae* for nutrient uptake and virulence, enabling the parasite to persist and proliferate within the host [125]. In conclusion, myxozoan parasites may exploit endocytosis to enter host cells and obtain essential nutrients, whereas the host may activate autophagy as a defense mechanism to mitigate stress and restrict infection—ultimately maintaining a balance between parasite survival and host protection.

Downregulation of muscle-related KEGG pathways such as cardiac muscle contraction, hypertrophic and dilated cardiomyopathy, and adrenergic signaling in cardiomyocytes, was observed in both *T. bryosalmonae* (Tb) and co-infected (Mc^+^) groups. Muscle contraction pathways were notably suppressed in co-infected gill tissue. Consistent with GO based findings, the KEGG pathways analysis also revealed downregulation of contractile genes such as *TNNT2* and *MYL3*, reinforcing infection-induced disruption of the muscular apparatus. The cardiac-specific *TNNT2* supports contractile function in gill tissue under stress [79]; its suppression here likely reflects structural remodeling or impaired contractile function. The downregulation of *MYL3*, a sarcomeric protein [126], was specific to the Mc^+^ group, indicating more severe tissue damage after the subsequent co-infection with *T. bryosalmonae*. Together, these transcriptomic changes point to infection-induced compromised gill perfusion, with co-infection exerting a more pronounced effect on both structural and physiological integrity.

The downregulated pathways in the transcriptomic data, such as DNA replication, ribosome biogenesis, and ECM-receptor interaction, suggest a coordinated shift from active cellular proliferation toward immune regulation, stress response, and structural remodeling. These findings are consistent with proteomic evidence of metabolic reprogramming and extracellular matrix alterations.

### 3.6. Integration with Previous Proteomic Findings

RNA-seq enables the detection of low-abundance genes, including novel isoforms and regulatory RNAs [127]. This allows for broad pathway analysis and offers deeper insights into immune regulation, particularly during early infection or co-infection. Compared to the proteomics-based investigation [20], our RNA-seq analysis provides a more comprehensive view of the host response to myxozoan infection. Using the same tissues as in our investigation, Saleh et al. (2024) conducted proteome profiling in rainbow trout following single and co-infections with *M. cerebralis* and *T. bryosalmonae* [20]. Key immunological markers such as *IFI44* and *C4B*, along with pathways including JAK-STAT and *IFN* signaling, were identified by both transcriptomic and proteomic approaches. As highlighted in the current study, these markers and pathways have also been reported in previous research on infections with both parasites. Therefore, the transcriptomic findings are consistent with the proteomic data in supporting the involvement of these immune components.

In comparison, a total of 47,152 genes were detected in our RNA-seq dataset after applying minimal expression thresholds, whereas the proteomic study by Saleh et al. [20] identified a comparatively small set of differentially regulated proteins, 16 in caudal fins and 27 in gills during single infections, and 4 and 11 proteins, respectively, during co-infection. The main reason for this difference is that RNA sequencing has a better sensitivity and wider dynamic range, with proteomic detection limited by things like quantity, extraction effectiveness, and ionization characteristics [128].

Furthermore, this discrepancy is also influenced by variations in data processing pipelines. Proteomics relies on peptide identification, which may restrict the detection of low abundance or tissue-specific proteins, while transcriptomic processes rely on direct sequence alignment to annotated genomes [129]. The existence of immune-related transcripts like *RSAD2*, *IFI44*, and *ISG15* indicates active host responses, even if post-transcriptional regulation, including mechanisms like nonsense-mediated degradation and microRNA interference, may cause some differences between transcripts and protein levels. These findings highlight that transcriptomic and proteomic approaches provide complementary insights into gene regulation, and transcriptomic data remain essential for identifying key molecular signatures during infection.

### 3.7. Overview of Immune Response

The caudal fin transcriptome profiling revealed distinct immune responses to both single and co-infections with *M. cerebralis* and *T. bryosalmonae*. *M. cerebralis* infection induced a strong innate immune response, marked by upregulation of complement factors, *IFN*-stimulated genes, cytokines, and vesicle trafficking components, along with partial suppression of adaptive immunity. In contrast, *T. bryosalmonae* infection elicited a moderate response involving *IFN* signaling, immune activation via metal ion sequestration, and autocrine signaling. Response to co-infection was strongly influenced by the sequence of infection. In fish infected with *M. cerebralis* followed by *T. bryosalmonae*, the response was dominated by stress-related gene expression, suggesting cortisol-mediated immunosuppression and metabolic stress. Conversely, Tb^+^ co-infection induced a markedly enhanced *IFN*-driven response, accompanied by suppression of DNA replication, ATP generation, and protein synthesis, indicative of immune overactivation, and tissue exhaustion. These findings demonstrate that the sequence of infection profoundly influences host immune dynamics, with Tb^+^ co-infection eliciting the most potent yet metabolically demanding response.

The transcriptomic analysis of the gill tissues revealed different immune responses during single and co-infections with *M. cerebralis* and *T. bryosalmonae*. *M. cerebralis* induced genes involved in early mucosal immune activation as well as antigen presentation pathways and cytotoxic T cell responses, indicating coordinated innate and adaptive immunity. While *T. bryosalmonae* single infection triggered an immune response focused on tissue repair and oxidative stress control, with controlled inflammation. Responses to co-infections varied by sequence of infection and were more complicated. In Mc^+^ (subsequent co-infection with *T. bryosalmonae*) fish, genes involved in epithelial maintenance, and anti-inflammatory signaling were upregulated, alongside T helper and regulatory cytokines, reflecting a balanced immune activation aimed at tissue homeostasis protection. However, downregulation of muscle function genes suggests a stress response after co-infection. In contrast, Tb^+^ fish (subsequent co-infection with *M. cerebralis*) exhibited a highly potent innate immune response, including substantial upregulation of *CRP* and *C3*, activation of *IL-1* signaling, and enhanced humoral immunity. Nevertheless, this was accompanied by suppression of neuronal signaling, antigen processing, and metabolic transport, indicating disrupted neuroimmune and metabolic homeostasis. These findings highlight the impact of infection sequence on mucosal immunity by demonstrating that co-infections, especially in Tb^+^, provoke a more intense but potentially dysregulated immune response in the gills.

**Figure 10:**
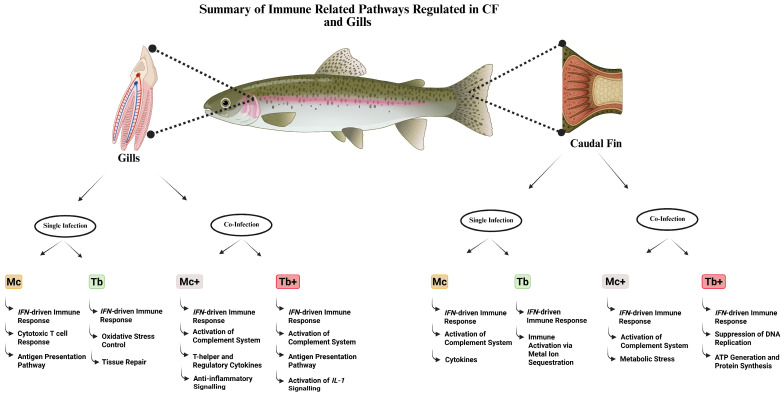
Summary of immune transcriptomic responses in caudal fin and gills of rainbow trout under single and co-infection conditions. The figure was created with BioRender.com.

Collectively, these findings describe gene expression patterns and offer important clues about how the host immune response dynamically adapts to different parasite loads and infection sequences at entry sites (Figure 10). In a previous study, the parasite loads were assessed by measuring the expression of RPL18 and 18S-rRNA, and a larger parasitic load of *T. bryosalmonae* was observed compared to *M. cerebralis* at the entry sites [20], which suggests faster replication of the parasite. The lack of shared upregulated genes between both parasites’ single infections in the current study is likely linked with the activation of distinct immune signaling pathways due to differences in tropism and infection dynamics. The robust *IFN* responses during Tb^+^ co-infection, while potentially protective, may drive immune overactivation and metabolic exhaustion. In contrast, the stress-related immunosuppression in Mc^+^ fish suggests reduced host immunity and increased susceptibility to secondary infections. These distinct immune pathways could serve as biomarkers of protective pathological responses and inform targeted strategies for co-infection management.

## 4. Materials and Methods

### 4.1. Experimental Design and Fish Sampling

Ninety-day-old, pathogen-free rainbow trout with a mean length of 4.02 ± 0.26 cm and a mean weight of 0.6 ± 0.15 g were housed in tanks (n = 3) with water maintained at 15 °C. Fish were fed daily at 1% of their body weight with floating trout pellets (Aqua Garant, Austria). For the exposure experiment, the North American strain (TL) of rainbow trout, known for its susceptibility to myxozoan parasites [130,131], was used. Before the exposure trial, a random sample of fish (n = 10) was tested using qPCR to confirm the absence of *M. cerebralis* and *T. bryosalmonae* [9]. Fish were monitored three times daily for signs of morbidity, such as erratic swimming, and any affected individuals were promptly removed and euthanized for sampling.

Initially, the fish were divided into three groups, following the experimental design previously described by Saleh et al. [20]. Briefly, fish (n = 36) in the first group (Mc) were exposed to *M. cerebralis* triactinomyxons (TAMs) at a dose of 2000 TAMs per fish. Fish (n = 36) in the second group (Tb) were exposed to *T. bryosalmonae* spores released from seven mature parasite sacs, with the exposure conducted at 16 °C [5]. The TAMs and spores were produced as previously described [37]. Thirty days post-exposure (dpe), half of the fish from the single-infection groups (Mc and Tb) were reciprocally co-infected with the other parasite, resulting in two co-infection groups: Mc^+^ (n = 18) and Tb^+^ (n = 18). An additional group of fish (n = 27) was used as a negative control and was exposed to a specific pathogen-free water. On day 31 post-infection, fish (n = 9) from each group and 3 biological replicates per sample were euthanized for sampling. Caudal fins (the portal of entry for *M. cerebralis*) and gills (the portal of entry for *T. bryosalmonae*) were rinsed with sterile phosphate-buffered saline and stored at −80 °C until further analysis. No infectious agents were detected prior to or during the experiment. In addition, viral screening was performed before and during the trial using BF-2 and EPC cell lines. Homogenates from the brain, whole viscera, spleen, and kidney were subjected to viral screening following standard cell culture procedures [132]. No viruses were detected before or during the experiment. Infections with *M. cerebralis* and/or *T. bryosalmonae* were confirmed in all relevant groups by qPCR during the in vivo phase, as previously described [9].

### 4.2. RNA Extraction, Library Preparation, and Sequencing

RNA was extracted from 31 days post-infection (1-day post-co-infection) caudal fin and gill samples. Briefly, total RNA was isolated from the gills (n = 15) and caudal fins (n = 15) of three fish per group (n = 5 groups) using the RNeasy Mini Kit (Qiagen, Hilden, Germany), following the manufacturer’s instructions. On-column DNase digestion was performed to eliminate residual genomic DNA. RNA concentration and quality were assessed using a Nanodrop 2000c spectrophotometer (Thermo Fischer Scientific, Wilmington, USA) and a 4200 TapeStation (Agilent Technologies, Santa Clara, CA, USA). All samples had RNA integrity numbers (RIN) above 8.0 and were used for cDNA library preparation.

For each sample, 500 ng of total RNA was used for mRNA purification with the Lexogen Poly(A) RNA Selection Kit V1.5. Library construction was then performed using the CORALL mRNA-Seq V2 Library Prep Kit (Lexogen, Vienna, Austria), following the manufacturer’s protocol. In total, 30 cDNA libraries (15 from caudal fin and 15 from gill tissue) were prepared. Library quality was assessed using the High Sensitivity D1000 ScreenTape Kit on the 4200 TapeStation System (Agilent Technologies). Sequencing was conducted on a NovaSeq 6000 system (Illumina, San Diego, CA, USA), generating 150 bp paired-end reads. Sequencing was carried out at the NGS unit of the Vienna Biocenter Core Facilities (VBCF, Vienna, Austria).

### 4.3. Mapping and Differential Expression Analysis of Host Genes

To prevent interference with downstream analyses, adapter sequences were removed using the Trimmomatic [133] software tool. Subsequent data analysis was performed using CLC Genomics Workbench 23.0.5 (Qiagen, Aarhus, Denmark). Raw sequencing reads underwent quality control assessment based on parameters including Phred score, read length, and the presence of adapter sequences [26]. Quality filtering was performed with an error probability threshold of 0.01, allowing a maximum of two ambiguous nucleotides per read. Bases with a Phred quality score ≤ 30 were removed and reads shorter than 50 nucleotides or longer than 150 nucleotides, as well as adapter and unique molecular identifier (UMI) sequences from library preparation, were discarded. Genome data for rainbow trout were obtained from the NCBI and used as a reference. Trimmed reads were mapped to the rainbow trout genome reference (USDA_OmykA_1.1, GCF_013265735.2) using the default parameters of the RNA-Seq tool in CLC Genomics Workbench (mismatch cost = 2; insertion cost = 3; deletion cost = 3; length fraction = 0.8; similarity fraction = 0.8). For differential expression, infected groups (Mc, Mc^+^, Tb, and Tb^+^) were compared pairwise to the respective non-infected control tissues.

Genes exhibiting a log_2_ fold change of >1 and a false discovery rate (FDR)-adjusted *p*-value < 0.01 were considered differentially expressed. Ortholog mapping for cross-species gene annotation was performed using g: Profiler [134]. Rainbow trout gene symbols were converted to human orthologs [24] due to the broader availability of annotation resources and biological knowledge for human genes compared to those of rainbow trout. Zebrafish orthologs were also identified; however, gene ontology (GO) term coverage for zebrafish was limited in comparison to human annotation. GO categories cellular component (CC), molecular function (MF), and biological process (BP), as well as Kyoto Encyclopedia of Genes and Genomes (KEGG) pathways enriched among annotated DEGs, were analyzed using FDR cut-off 0.05 in ShinyGO 0.80 [135].

### 4.4. Comparative Analysis with Proteomic Study

The consistency and integration of our transcriptomic findings were assessed by comparing the differentially expressed genes (DEGs) and enriched pathways with those reported in a recent proteomic study on *T. bryosalmonae* and *M. cerebralis* infections in rainbow trout [20]. Key immune pathways, genes, and tissue-specific responses were cross-referenced, with a focus on identifying both shared and distinct host responses in the caudal fins and gills.

### 4.5. Statistical Analysis

Differential gene expression analysis was conducted using the Differential Expression for RNA-Seq tool in CLC Genomics Workbench, which applies multifactorial statistical modeling based on a negative binomial Generalized Linear Model (GLM) and employs the TMM (trimmed mean of *M*-values) normalization method.

## 5. Conclusions

In summary, both caudal fin and gill tissues exhibited different but complementary immune modulation in single and co-infection with *M. cerebralis* and *T. bryosalmonae*. Single infections primarily activated a strong innate and a moderate adaptive immune response, which was more pronounced in *M. cerebralis* than in *T. bryosalmonae*. Co-infection triggered more complex and intense responses, with Tb^+^ consistently demonstrating enhanced *IFN* and complement-mediated immune activation in both tissues. This study provides helpful information about the tissue specific immune responses of rainbow trout under single and co-infection scenarios. This gene expression profiling offers potential biomarkers for early diagnosis and disease resistance and suggests targets for immune stimulants. These findings support improved disease monitoring and management practices in aquaculture, especially under complex pathogen exposure conditions.

## Figures and Tables

**Table 1:** Significantly enriched gene ontology terms in rainbow trout against single and coinfection in caudal fins.

Pathways	Regulation	Group	Pathway ID	Enrichment FDR	Pathway Genes	Fold Enrichment	nGenes	Genes
Antigen processing and presentation endogenous lipid antigen via MHC	Upregulated Pathways	Mc	GO:0048006	2.25 × 10^−8^	5	118.6	5	*CD1D*, *CD1A*, *CD1C*, *CD1B*, *CD1E*
Lipid antigen binding		Mc	GO:0030882	5.50 × 10^−9^	5	118.6	5	*CD1D*, *CD1A*, *CD1C*, *CD1B*, *CD1E*
Steroid catabolic process		Mc^+^	GO:0006706	4.27 × 10^−3^	26	135.4	2	*CYP24A1*, *CYP1A2*
Leukotriene-B4 20-monooxygenase activity		Mc^+^	GO:0050051	7.35 × 10^−7^	5	40.4	5	*CYP4F11*, *CYP4F2*, *CYP4F12*, *CYP4F3*, *CYP4A11*
Toll-like receptor 4 binding		Tb	GO:0035662	3.40 × 10^−4^	4	309.2	2	*S100A8*, *S100A9*
RAGE receptor binding		Tb	GO:0050786	6.27 × 10^−8^	10	247.4	4	*S100A4*, *S100A8*, *S100A7*, *S100A9*
Positive reg. of toll-like receptor 7 signaling pathway		Tb^+^	GO:0034157	3.96 × 10^−2^	6	476.7	1	*RSAD2*
Lipid antigen binding		Tb^+^	GO:0030882	3.441 × 10^−6^	5	31.4	5	*CD1D*, *CD1A*, *CD1C*, *CD1B*, *CD1E*
Carnitine transmembrane transporter activity	Downregulated Pathways	Mc	GO:0015226	1.06 × 10^−3^	4	215.9	2	*SLC22A5*, *SLC22A4*
Cellular response to fluid shear stress		Mc	GO:0071498	4.50 × 10^−3^	20	152.5	2	*PTGS2*, *KLF4*
MCM complex		Mc^+^	GO:0042555	1.76 × 10^−8^	11	31.8	7	*MCM6*, *MCM9*, *MCM3*, *MCM7*, *MMS22L*, *TONSL*, *MCMBP*
Nuclear replication fork		Mc^+^	GO:0043596	5.02 × 10^−13^	38	18.4	14	*POLA2*, *MCM10*, *POLA1*, *POLD2*, *TIMELESS*, *RPA2*, *MMS22L*, *PRIM1*, *WDHD1*, *PRPF19*, *PCNA*, *SMARCA5*, *TONSL*, *MCM3*
Elastin catabolic proc.		Tb	GO:0060309	3.98 × 10^−2^	4	715.0	1	*MMP12*
Endothelial cell–cell adhesion		Tb	GO:0071603	3.98 × 10^−2^	4	715.0	1	*THBS4*
Leukotriene-B4 20-monooxygenase activity		Tb^+^	GO:0050051	9.46 × 10^−7^	5	36.5	5	*CYP4F11*, *CYP4F2*, *CYP4F12*, *CYP4F3*, *CYP4A11*
DNA polymerase complex		Tb^+^	GO:0042575	1.115 × 10^−6^	20	14.6	8	*POLA2*, *POLE2*, *POLA1*, *POLD2*, *PRIM1*, *DNA2*, *POLG*, *MCM3*

**Table 2:** Summary of significantly regulated KEGG pathways in infected caudal fins.

Sr No.	Pathways	Group	Fold Enrichment	Pathway ID	No. of Identified Genes	Genes
Upregulated Pathways						
1	JAK-STAT signaling pathway	Mc	8.049990405	hsa04630	11	*IL23R*, *GHR*, *IL2RG*, *IL3RA*, *IL6R*, *IL11RA*, *IL12RB1*, *IL13RA1*, *IL17D*, *CRLF2*, *OSMR*
2	Chemokine signaling pathway	Mc	6.207036866	hsa04062	10	*CCL26*, *CCL1*, *CCL2*, *CCL7*, *CCL8*, *CCL11*, *CCL13*, *CCL22*, *CCL24*, *CX3CL1*
3	Th17 cell differentiation	Mc	5.488629822	hsa04659	5	*IL23R*, *TBX21*, *IL2RG*, *IL6R*, *IL12RB1*
4	Autophagy-animal	Mc^+^	4.865435851	hsa04140	17	*GABARAPL2*, *CTSL*, *ATG14*, *ATG4B*, *ATG9B*, *PIK3R4*, *SH3GLB1*, *PDPK1*, *ZFYVE1*, *PRKAA2*, *TP53INP2*, *RAB1A*, *DEPTOR*, *RAB7A*, *ATG9A*, *IRS4*, *ULK2*
5	JAK-STAT signaling pathway	Mc^+^	3.487425697	hsa04630	14	*IL23R*, *GHR*, *IL2RG*, *IL3RA*, *IL6R*, *IL11RA*, *IL12RB1*, *IL13RA1*, *IL13RA2*, *CRLF2*, *STAT5A*, *STAT5B*, *SOCS2*, *OSMR*
6	*IL-17* signaling pathway	Tb	26.59808195	hsa04657	4	*S100A7A*, *S100A7*, *S100A8*, *S100A9*
7	JAK-STAT signaling pathway	Tb^+^	4.656288156	hsa04630	24	*SOCS4*, *IL23R*, *GHR*, *IFNAR1*, *IFNAR2*, *IFNGR1*, *IL2RG*, *IL3RA*, *IL6R*, *IL10RB*, *IL11RA*, *IL12RB1*, *IL13RA1*, *JAK1*, *JAK2*, *PDGFRB*, *IL17D*, *CRLF2*, *STAT1*, *STAT2*, *STAT5A*, *STAT5B*, *SOCS2*, *OSMR*
Downregulated Pathways						
8	Protein processing in endoplasmic reticulum	Mc^+^	6.457418086	hsa04141	30	*PDIA6*, *SEC23B*, *CKAP4*, *DAD1*, *EIF2S1*, *STT3B*, *SEC31A*, *DNAJC5G*, *SEC61A1*, *HSPA2*, *HSPA5*, *HSP90AA1*, *STT3A*, *LMAN1*, *P4HB*, *DNAJC10*, *UGGT2*, *DNAJC3*, *MAN1C1*, *RPN1*, *RPN2*, *RRBP1*, *DNAJC1*, *SSR4*, *HSP90B1*, *VCP*, *CALR*, *TXNDC5*, *CAPN2*, *PLAA*
9	DNA replication	Mc^+^	14.14652888	hsa03030	14	*DNA2*, *FEN1*, *POLA2*, *LIG1*, *MCM3*, *MCM6*, *MCM7*, *PCNA*, *POLA1*, *POLD2*, *POLE2*, *PRIM1*, *RFC2*, *RPA2*
10	Ribosome biogenesis in eukaryotes	Mc^+^	7.558813206	hsa03008	16	*EMG1*, *UTP14A*, *POP1*, *WDR36*, *DKC1*, *WDR43*, *REXO2*, *EIF6*, *SNU13*, *GAR1*, *HEATR1*, *NAT10*, *UTP6*, *PWP2*, *NOL6*, *UTP14C*
11	ECM-receptor interaction	Tb	32.50142045	hsa04512	1	*THBS4*
12	TNF signaling pathway	Tb	25.53683036	hsa04668	1	*MMP3*
13	DNA replication	Tb^+^	15.2053429	hsa03030	15	*DNA2*, *FEN1*, *POLA2*, *LIG1*, *MCM3*, *MCM6*, *MCM7*, *PCNA*, *POLA1*, *POLD2*, *POLE2*, *PRIM1*, *RFC2*, *RFC4*, *RPA2*
14	Protein processing in endoplasmic reticulum	Tb^+^	3.886809547	hsa04141	18	*SEC23B*, *DAD1*, *EIF2S1*, *STT3B*, *SEC31A*, *SEC61G*, *SEC61A1*, *HSPA2*, *HSP90AA1*, *LMAN1*, *MAN1C1*, *RPN1*, *RPN2*, *RRBP1*, *DNAJC1*, *SSR4*, *CALR*, *TXNDC5*

**Table 3:** Significantly enriched gene ontology terms in rainbow trout against single and coinfection in gills.

Pathways	Regulation	Group	Pathway ID	Enrichment FDR	Pathway Genes	Fold Enrichment	nGenes	Genes
Antigen processing and presentation endogenous lipid antigen via MHC	Upregulated Pathways	Mc	GO:0048006	3.98 × 10^−12^	5	762.7	5	*CD1D*, *CD1A*, *CD1C*, *CD1B*, *CD1E*
Exogenous lipid antigen binding		Mc	GO:0030884	1.86 × 10^−13^	5	762.7	5	*CD1D*, *CD1A*, *CD1C*, *CD1B*, *CD1E*
Collagen type IX trimer		Mc^+^	GO:0005594	2.04 × 10^−3^	3	111.3	2	*COL9A3*, *COL9A2*
CCR3 chemokine receptor binding		Mc^+^	GO:0031728	4.36 × 10^−5^	5	100.2	5	*CCL26*, *CCL24*, *CCL11*
Integrin alpha4-beta1 complex		Tb	GO:0034668	4.40 × 10^−2^	3	401.4	1	*ITGA4*
Interleukin-8 receptor binding		Tb	GO:0005153	3.76 × 10^−2^	3	401.4	1	*CX3CL1*
Alcohol dehydrogenase activity zinc-dependent		Tb^+^	GO:0004024	2.09 × 10^−11^	7	63.0	7	*ADH6*, *ADH1A*, *ADH7*, *ADH1B*, *ADH5*, *ADH4*, *ADH1C*
Humoral immune response mediated by circulating immunoglobulin		Tb^+^	GO:0002455	1.03 × 10^−3^	67	60.3	3	*CRP*, *APCS*, *C3*
Negative reg. of synaptic plasticity	Downregulated Pathways	Mc	GO:0031914	7.65 × 10^−3^	3	3813.5	1	*UNC13C*
Dense core granule priming		Mc	GO:0061789	7.65 × 10^−3^	3	3813.5	1	*UNC13C*
Cardiac Troponin complex		Mc^+^	GO:1990584	3.06 × 10^−2^	3	401.4	1	*TNNT2*
Myosin II heavy chain binding		Mc^+^	GO:0032038	22.99 × 10^−2^	3	401.4	1	*MYL3*
Cardiac Troponin complex		Tb	GO:1990584	1.70 × 10^−3^	3	7627.0	1	*TNNT2*
Regulation of muscle filament sliding		Tb	GO:0032971	1.59 × 10^−2^	4	5720.3	1	*TNNT2*
Immature B cell differentiation		Tb^+^	GO:0002327	1.37 × 10^−2^	13	185.3	2	*RAG1*, *RAG2*
Integrator complex		Tb^+^	GO:0032039	3.05 × 10^−11^	30	18.2	12	*INTS6L*, *SAGE1*, *CT45A5*, *CT45A1*, *CT45A3*, *CT45A10*, *CT45A9*, *CT45A2*, *CT45A7*, *CT45A8*, *CT45A6*, *INTS14*

**Table 4:** Summary of significantly regulated KEGG pathways in infected gills.

Sr No.	Pathways	Group	Fold Enrichment	Pathway ID	No. of Identified Genes	Genes
Upregulated Pathways						
1	JAK-STAT signaling pathway	Mc^+^	12.37	hsa04630	12	*SOCS4*, *CSF3R*, *IL23R*, *GHR*, *IL2RG*, *IL3RA*, *IL6R*, *IL11RA*, *IL12RB1*, *IL13RA1*, *CRLF2*, *OSMR*
2	*IL-17* signaling pathway	Mc^+^	10.77	hsa04657	6	*IL17RA*, *IL1B*, *PTGS2*, *CCL2*, *CCL7*, *CCL11*
3	Th17 cell differentiation	Mc^+^	7.73	hsa04659	5	*IL23R*, *IL1B*, *IL2RG*, *IL6R*, *IL12RB1*
4	TNF signaling pathway	Mc^+^	7.45	hsa04668	5	*IL1B*, *PTGS2*, *BCL3*, *CCL2*, *CX3CL1*
5	Natural killer-cell-mediated cytotoxicity	Tb	18.52	hsa04650	2	*ITGB2*, *PRF1*
6	JAK-STAT signaling pathway	Tb^+^	5.23	hsa04630	21	*CISH*, *SOCS4*, *CSF3R*, *IL23R*, *EGFR*, *GHR*, *IL2RB*, *IL2RG*, *IL3RA*, *IL6R*, *IL10RB*, *IL11RA*, *IL12RB1*, *IL13RA1*, *JAK1*, *CRLF2*, *STAT1*, *STAT2*, *STAT3*, *SOCS2*, *OSMR*
7	Cytokine–cytokine receptor interaction	Tb^+^	5.21	hsa04060	38	*CCL26*, *IL31RA*, *CSF1R*, *CSF3R*, *IL23R*, *IL17RA*, *IL36RN*, *GHR*, *IL36B*, *IL37*, *IL36A*, *FAS*, *IL1B*, *IL1RN*, *IL2RB*, *IL2RG*, *IL3RA*, *IL6R*, *IL10RB*, *IL11RA*, *IL12RB1*, *IL13RA1*, *IL36G*, *CCL1*, *CCL2*, *CCL7*, *CCL8*, *CCL11*, *CCL13*, *CCL22*, *CCL24*, *CX3CL1*, *CRLF2*, *CXCR4*, *IL1F10*, *TNFSF14*, *TNFSF10*, *OSMR*
Downregulated Pathways						
8	Hypertrophic cardiomyopathy	Tb	254.23	hsa05410	1	*TNNT2*
9	Dilated cardiomyopathy	Tb	238.34	hsa05414	1	*TNNT2*
10	Hypertrophic cardiomyopathy	Mc^+^	53.52	hsa05410	4	*MYL3*, *ATP2A1*, *TGFB3*, *TNNT2*
11	Dilated cardiomyopathy	Mc^+^	50.17	hsa05414	4	*MYL3*, *ATP2A1*, *TGFB3*, *TNNT2*
12	Cardiac muscle contraction	Mc^+^	41.52	hsa04260	3	*MYL3*, *ATP2A1*, *TNNT2*
13	DNA replication	Tb^+^	18.91	hsa03030	15	*DNA2*, *FEN1*, *POLA2*, *LIG1*, *MCM3*, *MCM6*, *MCM7*, *PCNA*, *POLA1*, *POLD2*, *POLE2*, *PRIM1*, *RFC2*, *RFC4*, *RPA2*
14	Protein processing in the endoplasmic reticulum	Tb^+^	5.104008171	hsa04141	19	*CKAP4*, *DAD1*, *EIF2S1*, *STT3B*, *SEC31A*, *SEC61A1*, *HSP90AA1*, *STT3A*, *LMAN1*, *UGGT2*, *RPN1*, *RPN2*, *RRBP1*, *DNAJC1*, *SSR4*, *VCP*, *CALR*, *TXNDC5*, *PLAA*

## Data Availability

The original contributions presented in this study are included in the article/supplementary material. Further inquiries can be directed to the corresponding author.

## Supplementary Materials

The following supporting information can be downloaded at https://www.mdpi.com/article/10.3390/ijms26178148/s1.

## Abbreviations

WD	Whirling Disease
PKD	Proliferative Kidney Disease
TAMs	Triactinomyxon Spores
*SOCS*	Suppressor of Cytokine Signaling
STAT	Signal Transducer and Activator of Transcription
CNTF	Ciliary Neurotrophic Factor Receptor Activity
GO	Gene Ontology
KEGG	Kyoto Encyclopaedia of Genes and Genomes
CF	Caudal Fins
HSP	Heat Shock Protein
MCP	Monocyte Chemotactic Protein
PAS	Phagophore Assembly Site
TNF	Tumor Necrosis Factor
*IFI44*	Interferon Induced Protein 44
*RSAD2*	Radical S-adenosyl Methionine Domain Containing 2
*ISG15*	ISG15 Ubiquitin-Like Modifier
*TLR7*	Toll-Like Receptor 7
